# Key features of illness and treatment experiences in longstanding anorexia nervosa: qualitative descriptive study

**DOI:** 10.1192/bjo.2025.10923

**Published:** 2025-12-22

**Authors:** Laura Kiely, Phillipa Hay, Janet Conti

**Affiliations:** School of Medicine, Western Sydney University, Campbelltown, NSW, Australia; Translational Health Research Institute, Western Sydney University, Penrith, NSW, Australia; Mental Health Services, Camden and Campbelltown Hospitals, SWSLHD, Campbelltown, NSW, Australia; School of Psychology, Western Sydney University, Penrith, Australia

**Keywords:** Anorexia nervosa, self-report, quality of life, surveys and questionnaires, iatrogenic disease

## Abstract

**Background:**

Studies have consistently found that up to 20% of people with anorexia nervosa experience a persistent illness, resulting in considerable psychosocial impairment, morbidity and mortality. This has been variously termed severe and enduring anorexia nervosa or longstanding anorexia nervosa (L-AN). Conflicting findings have hindered progress in distinguishing the nosological features of individuals with persistent illness.

**Aims:**

This study aims to investigate the putative defining features of individuals reporting symptoms of L-AN, including consideration of their treatment trajectory.

**Method:**

This cross-sectional study, drawing from a mixed-methods design, utilised a sample of symptomatic individuals who reported experiencing eating disorder treatment (*n* = 208). Several qualitative and quantitative data strands (a–c) were embedded within a single, self-report questionnaire measuring eating disorder severity and treatment experiences. Between-group comparisons were used to compare those of shorter (<3 years) and longer (>7 years) duration of illness.

**Results:**

No between-group differences were found in measures of severity, including body mass index (kg/m^2^), eating disorder symptom scores, psychological distress or perceived health-related quality of life. However, those with L-AN had a significantly higher number of mental and physical health comorbidities, longer treatment delay, greater number of episodes of treatment and poorer subjective ratings of their treatment experiences.

**Conclusions:**

Delineating L-AN by severity may be inappropriate; anorexia nervosa of any duration is a severe illness. This study suggests that treatments, or lack thereof, may have an inadvertent impact on duration of illness. Future focus needs to be on reconceptualising L-AN and its treatments. Treatment refinements informed by lived experience are proposed.

Significant progress has been made in developing effective treatments for people with anorexia nervosa, including out-patient cognitive–behavioural therapy (CBT)^1^ and other psychological therapies.^2^ However, when defining recovery in terms of eating disorder symptoms and body mass index (BMI, kg/m^2^), outcome studies have repeatedly shown that recovery in the first 10 years of illness is considered the exception, and that at least 20% of people experience persistent illness.^3,4^ Understanding how treatments work (mechanism-based studies) and capturing the impact of therapeutic effects are highlighted as fundamental to future treatment innovation.^2^ For those with persistent illness, there is considerable psychosocial impairment and morbidity.^5,6^ This form of anorexia nervosa has variously been termed severe and enduring (SE-AN) or longstanding (L-AN), with some evidence suggesting that the latter term is favoured by people with lived experience.^a,7,8^ The conceptualisation of L-AN is nascent, with limited research having been conducted, and the proposed criteria are subject to current debate.^9,10^ It is essential to understand the illness experience to progress the conceptualisation of L-AN, and to understand treatment experiences to inform the development of more responsive and personalised treatments.^11–16^

## Concept of SE-AN

The concept of a ‘severe and enduring’ eating disorder (SEED) was first proposed in 2009 by Robinson^17^ and, since then, has been most extensively studied in people with anorexia nervosa (SE-AN). The meaningfulness of the SEED/SE-AN construct divides opinion, most notably in conflation of the ‘SE-AN’ staging label with ‘hopelessness’, where people with lived experience report feeling abandoned by healthcare based on a label that is not empirically defined.^10,18–20^ Clinician researchers have echoed these concerns by pointing out that those with the most need may receive the least help.^21^ This represents a misuse of the diagnostic intention of SE-AN, which was foremost to assist in identifying features of a subgroup of people with anorexia nervosa to inform theoretical conceptualisations and novel treatments.^22^ Recognising a persistent state of illness that has eluded multiple treatment efforts can also open clinical discussions around alternative approaches to care. This includes emphasis on the principles of recovery-orientated care,^23,24^ as described for eating disorder treatment (e.g. ref.^25^), encompassing several key components, namely (a) holding optimism in recovery, including improved well-being as well as improved symptoms; (b) maintaining a focus on empowerment (maximal self-determination and self-management); and (c) engagement in a meaningful life (life quality), while recognising the dignity of risk, offering maximal choice and promoting positive risk-taking and safety. Centring quality of life (QoL) is not universally accepted for people with eating disorders, even though such an approach has been shown to markedly improve treatment engagement and ‘paradoxically’ improve symptoms.^26–28^

## Defining the features of L-AN

Attempts to distinguish the defining features of a subgroup of people for whom anorexia nervosa persists, despite the treatment they have received, are problematic. Conflicting research regarding putative features such as severity, duration and QoL highlights these complexities. Concerning the most widely agreed-upon feature of duration, this varies between 3 and up to 10 years,^29–31^ with mixed results regarding the duration of illness and treatment response.^4,9,32–39^ Complicating the picture are overlooked clinical features such as identity disturbance in the conceptualisation of anorexia nervosa.^40^ There is also the consideration that treatments (or lack thereof) may inadvertently cause harm (i.e. be iatrogenic) and contribute to a persistent illness trajectory.^40–42^ Duration is important, but it appears that the length of time determining a ‘long’ duration is uncertain, and duration alone is insufficient to delineate a L-AN subgroup.^6,9^

The concept of severity is also problematic as a defining feature of L-AN. The Diagnostic and Statistical Manual (DSM-5)^43^ applies BMI alone as a severity marker. However, BMI was neither found to predict symptom severity^9,44^ nor to be a central feature in anorexia nervosa in one network analysis.^45^ Clinically, BMI has limited helpfulness in guiding treatment decisions regarding stepping up to in-patient care,^46^ and eating disorder symptoms and behaviours persist following weight restoration.^47^ Notably, however, both low BMI and duration of illness (DoI) have been reported to predict mortality,^48^ and low BMI at treatment discharge is a strong predictor of relapse^49^ and poorer outcomes.^11^ Thus, while BMI is important to consider clinically, other indicators of severity, such as eating disorder symptoms and behaviours, distress and impairment (QoL and psychosocial functioning), may advance the conceptualisation of SE-AN.^38,40^

In response to these ambiguities, one group conducted a secondary analysis of a recent, 22-centre out-patient study and delineated outcomes (BMI, eating disorder symptoms, work and social adjustment and distress (depression and anxiety)) in a sample of 187 people (97% women) with anorexia nervosa and who received endorsed, evidence-based treatments.^38^ Participants were categorised by ‘early stage’ (ES-AN; <3 years) and ‘severe and enduring’ (68%), applying a delineator of >7 years and high distress. At 12-month follow-up, the main findings confirmed a staging model for anorexia nervosa^50–52^ whereby higher rates of service utilisation, poorer work/social adjustment and higher eating disorder symptoms were observed temporally in the SE-AN group.

## Comorbidity and psychosocial determinants

Comorbidity is the norm for people with anorexia nervosa.^45,53^ Register studies that permit large population-based information reveal that psychiatric comorbidities alongside anorexia nervosa predict poorer health outcomes.^54^ The directionality of the relationship between anorexia nervosa and comorbidity is unclear,^55^ whereby comorbid psychiatric symptoms can predate the eating disorder, persist with illness remission and worsen outcomes.^32,56–58^ Further research is needed.^59,60^ Recent research on psychosocial determinants (trauma/post-traumatic stress disorder (PTSD)) affecting outcomes and neurodiversity as complicating factors^61,62^ has yet to be synthesised into meaningful findings.^1^ For instance, the implications of co-occurring autism on the transition to L-AN is not yet known. Several authors have suggested that QoL in L-AN is the most consistent marker of severity^9,30,38^ and is potentially one unified delineator. In eating disorders, a longer illness severely impacts a person’s physical and mental health-related quality of life,^40^ and improvement in QoL is central to both eating disorder symptom improvement and pathways to recovery.^63^

## Treatment and illness persistence

In considering conceptualisations of L-AN, a further putative and important feature is the impact of treatments. Most studies testing the SE-AN concept have omitted information about participants’ prior treatment(s). Notably, the current efficacy of evidence-based treatments for anorexia nervosa is modest at best.^64^ This has led some authors to criticise current conceptualisations of anorexia nervosa which, in turn, inform the theoretical bases of treatments as reductionistic.^65^ The duration of untreated eating disorder (DUED) is a complicating factor in persistence, where longer DUED is associated with poorer outcomes.^33^ Recent qualitative literature has contributed to the understanding of people’s experiences of treatment, and has uncovered repeated unhelpful and, at worst, harmful episodes of care in those with L-AN, with increasing reports of treatment-related trauma and PTSD.^28,41,66,67^ Some have posited L-AN as being a ‘fertile ground for iatrogenic development’,^68^ and the impacts of perceived unhelpful treatment experiences are yet to be quantified.^40^

Nonetheless, treatment delay is implicated in persistent illness^33,46,69^ and longer treatment duration is protective, i.e. associated with lower mortality.^3,4,11^ Maintaining engagement with healthcare is a priority, and medical advances in the management of eating disorders have facilitated the safer delivery of less restrictive care to improve the QoL and longevity of those with L-AN.^46^

## L-AN criteria for empirical testing

Bamford and Mountford^31^ were among the first to propose testable criteria for L-AN and in 2018, based on growing interest in the clinical and research utility of the SE-AN construct, Hay and Touyz^29^ proposed three criteria: (a) a persistent state of dietary restriction, underweight and overvaluation of weight/shape with functional impairment; (b) duration of >3 years of anorexia nervosa; and (c) exposure to at least two evidence-based treatments, appropriately delivered together with a diagnostic assessment and formulation that incorporates an assessment of the person’s eating disorder health literacy and stage of change. The second criterion, related to duration, applies the shortest duration known to be associated with a persistent illness, and this has subsequently been criticised;^70,71^ a duration of 7 years or longer is more likely to be used.^38^

## Study aims

To our knowledge, no studies have collectively considered and tested the validity of the criteria of Hay and Touyz,^29^ or any similar criteria, particularly the last of these (treatment exposure). Thus, the present study aimed to first identify and describe a community sample of individuals who report being (a) currently symptomatic for anorexia nervosa and (b) with at least 3 years’ experience of anorexia nervosa and its treatment; or, in other words, to explore face validity.^72^ Second, compared with those of shorter illness duration (≤3 years), we hypothesised that those of long duration (≥7 years) will have poorer health-related QoL (HR-QoL), more severe depression and anxiety, longer treatment latency and a greater number of comorbidities and treatment episodes, thus investigating descriptive validity.^73^ Based on concurrent research,^28,40,69^ a further aim was to compare the sense of self and identity in those with longer and shorter durations. The quantity and experiences of participants’ treatment experiences were also qualitatively explored in relation to their illness trajectories. Hence, this research aimed to investigate the face validity of the L-AN construct and explore the descriptive validity of concurrent validators (such as associations with comorbidities), as distinct from early-stage anorexia nervosa, to determine whether L-AN may inform a different approach within treatment. Complex questions require complex methodologies to capture multiple layers of evidence, and therefore a mixed-methods, sequential design was utilised.^73^

## Method

### Study design

In the present cross-sectional survey study, a pragmatist paradigm^74^ informed a triphasic, embedded-sequential, mixed-methods design^75^ (see Supplementary 1 available at https://doi.org/10.1192/bjo.2025.10923). This design aimed to synergise qualitative and quantitative findings, emphasising complementarity, completeness and explanation,^76^ to build a dimensional understanding of the L-AN experience, including the impact of treatments. Consistent with this research method, study results are reported in a manner that allows the qualitative extension of quantitative findings, and vice versa. The Consolidated Criteria for Reporting Qualitative Research (COREQ) checklist^77^ and the Strengthening the Reporting of Observational Studies in Epidemiology statement^78^ for cross-sectional studies were considered, and are appended (see Supplementary 2).

### Participants

People in the community aged ≥16 years, and who self-reported ever receiving treatment for a reported diagnosis of anorexia nervosa, were included as participants. There were no specific exclusion criteria but, to complete the survey, there was a presumption of computer literacy in the English language. The final sample consisted of 208 adults, mostly women (193 of 208, 93%) with 7% (15 of 208) male or non-binary, from 6 countries, predominantly Australia (75 of 202, 37%), the USA (97 of 202, 39%) and the UK (31 of 202, 15.3%). This corresponded with a homogenous White/European ethnic distribution, whereby a minority (32 of 202, 16%) identified as Asian, Maori/Pacific Islander or Aboriginal/Torres Strait Islander.

### Survey instrument

A comprehensive, 30-min, online, self-report ‘Eating Disorder and Treatment Experience Survey’ (EDTES), previously developed by researchers J.C. and P.H.,^79^ included both open- and closed-ended questions, providing both qualitative and quantitative data. The survey collected demographic and clinical data, including open-ended qualitative questions on treatment experiences, as well as validated measures of current eating disorder symptoms and mood.^80^ Closed-ended questions were applied using the Treatment Experiences Sliding Scale Rater (TESSR),^79^ also developed in 2018 by researchers J.C. and P.H. EDTES was adapted (data-set b) by L.K., in conjunction with J.C. and P.H., for use in the present 2022 study. New additions to the survey in 2022 included two validated instruments for measurement of HR-QoL^81^ and sense of self,^82^ as well as further questions regarding clinical history (e.g. physical and psychiatric comorbidities). The two data-sets (a and b) were combined to provide sufficient statistical power for analysis.

#### Survey measures

The survey utilised five validated instruments. The Eating Disorder Examination Questionnaire (Short) (EDE-QS) is a 12-item eating disorder-specific self-report measure that utilises a 4-point frequency scale of 0–3 related to a respondent’s previous 7 days.^83^ The Self-Concept and Identity Measure (SCIM)^82^ considers a person’s identity functioning across three subdomains: lack of identity, consolidated identity and disturbed identity. The Health-Related Quality of Life – Short Form 12 version 2 (SF12v2)^81^ measures the perceived impact of both mental and physical health components on QoL, using a 5-point Likert scale. The Hospital Anxiety and Depression Score (HADS)^84^ is a self-report measure used to screen for acute levels of depression and anxiety across 14 questions, with a score range of 0–21 for each. The TESSR^79^ is designed to explore participants’ self-identified least and most helpful experiences with eating disorder treatment. Questions are rated using a Likert sliding-scale response (−50 to 50, range 100) in various domains. See Supplementary 3 for further descriptions, validity and internal consistency of these instruments.

### Key variables

#### DUED

DUED was calculated using the difference (years) between the self-reported age of onset of the first symptoms of an eating disorder and that of the first treatment episode.

#### DoI

The parameter DoI was self-reported in response to a question about how long the person perceived they had experienced symptoms of an eating disorder. To explore between-group differences for DoI, time periods were based on (albeit very limited) previous research. Ambwani et al^38^ categorised participants as either ES-AN (if the duration was ≤3 years) or L-AN, by applying a delineator of ≥7 years. The period of 3 years has also been found to be associated with a better outcome in early studies of family therapy for anorexia nervosa.^85^ The most frequently used duration for a long duration is 7 years, and this was the measure employed in the only randomised controlled trial of therapy for adults with L-AN.^26^

#### Total number of care episodes

The total number of care episodes comprised the sum of self-reported in-patient/high-level admissions (e.g. residential eating disorder treatment, specialised eating disorder unit or mental health unit) and the number of separate times that out-patient psychological therapy (e.g. family therapy and/or individual therapy) had been initiated.

### Qualitative thematic analyses of treatment experiences, self-concept and identity and open-ended survey questions

**Fig. 1:**
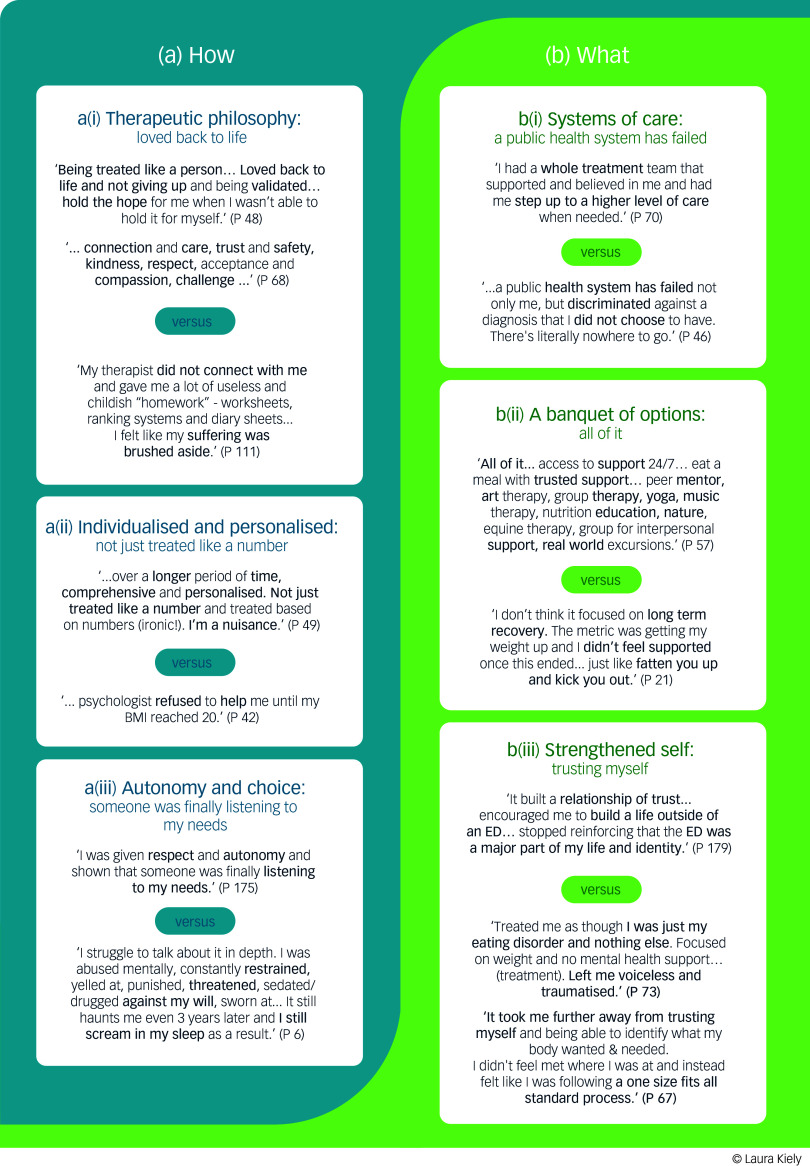
Thematic analysis: treatment needs of those with longstanding anorexia nervosa. P, participant number; BMI, body mass index; ED, eating disorder.

Participants were asked several open-ended questions to supplement two of the quantitative measures about their treatment experiences (TESSR) and sense of self (SCIM) (see Supplementary 4). An inductive reflexive thematic analysis^86^ was used to generate themes from all participant responses to these sets of open-ended questions from TESSR and SCIM. An iterative methodology (see Supplementary 4) was applied to each question from each respondent and therefore, by including and coding all responses, sufficiency and ‘information power’ were addressed.^87^ The final phase involved translating the analyses into a visual infographic for the TESSR analysis (Fig. 1 (see Results section)) and a summary table for the SCIM analysis (Supplementary 4 (see Results section)), thereby communicating the findings. These two analyses support the interpretations of quantitative findings contemporaneously.

### Authors’ reflective statements

By way of reflexivity, and in response to domain 1 of COREQ^77^ and enhancing validity, all authors contributed a reflective statement (see Supplementary 5).

### Ethics, consent, distribution, data collection and analyses

The study was approved by Western Sydney University’s ethics committee (approval no. H14856). The survey was designed and disseminated using Qualtrics software (2002 for Windows; Qualtrics, LLC, USA; https://www.qualtrics.com/). Recruitment utilised snowballing, which involves connecting potential participants through shared social networks.^88^ This was initiated by advertising through key international eating disorder organisations and personnel (e.g. Eating Disorders Victoria and the Carolyn Costin Institute). Facebook and Instagram were used as communication platforms, in conjunction with hard-copy posters featuring quick-response codes, each stipulating specific inclusion criteria. Data collection proceeded for 6 months (July–December 2022), with active advertising and recruitment in the first month. Participants provided written informed consent to participate in the study. Information about the statistical plan is available in Supplementary 3.

## Results

### Participant clinical characteristics

The present study participants were a symptomatic sample with a mean EDE-QS score of 18.4 (s.d. 7.5), and all participants scored above 1 s.d. below the mean of a clinical sample as reported by Gideon et al.^83^ Participant characteristics are summarised in Table 1.

**Table 1:** Participant demographics for all participants identifying experience as anorexia nervosa (total *n* = 208)

Parameters	Respondents (*n*)^a^	Statistic
		Mean (s.d.)
Eating Disorder Examination Questionnaire (Short)	208	18.4 (7.5)
Age of eating disorder symptom onset (years)	158	14.4 (4.4)
Current body mass index (kg/m^2^)	148	20.5 (4.8)
Hospital Anxiety and Depression Scale (anxiety)	205	10.1 (4.3)
Hospital Anxiety and Depression Scale (depression)	205	9.6 (3.5)
Health-Related Quality of Life – Short Form 12 mental health component score	74	34.5 (11.3)
Health-Related Quality of Life – Short Form 12 physical health component score	74	48.0 (12.6)
		Median (IQR)
Age (years)	201	24.0 (19–32)
Duration of eating disorder (years)	199	8.0 (4.0–15.0)
Number of mental health comorbidities	66	3.0 (2–4)
Number of physical health comorbidities	68	3.0 (1–5)
		*n* (%)
Additional mental health diagnosis	72	66 (92)
Self-reported comorbid diagnosis		
Anxiety/panic	67	54 (80.6)
Depression	66	50 (75.8)
Obsessive–compulsive disorder	67	23 (34.3)
Post-traumatic stress disorder	67	24 (35.8)
Neurodevelopmental (ADHD/autism)	66	16 (24.2)
Substance use disorder	66	12 (18.2)
Sleep disorder	66	14 (21.2)
Other (dissociative, personality and/or bipolar disorders, other addictions, psychosis)	66	32 (48.5)
Psychiatric experiences		
Self-harm	66	33 (50.0)
Suicidal ideation	67	33 (49.3)
Abuse/neglect (childhood)	67	24 (35.8)
Sexual abuse	67	19 (28.4)

IQR, interquartile range; ADHD, attention-deficit hyperactivity disorder.

a. There are different *n* values in this table because (a) additional questions were added to the survey in 2022 and (b) not all participants answered all survey questions.

The age of onset was early adolescence, and most people had a long DoI. The mean BMI (kg/m^2^) was 20.5 (i.e. low normal), and a high proportion reported anxiety/panic disorder, PTSD and depression. In addition, experiences of self-harm and suicide ideation occurred in half of all people, and approximately a third reported sexual abuse, childhood abuse and/or neglect. Of the 72 participants who reported an additional mental health diagnosis, the median number of comorbidities was 3 (interquartile range (IQR) 2–4). The majority (59 of 66, 89%) of people reported receiving specific treatment for comorbidity, most commonly for depression (73%), anxiety (68%) and ‘trauma’ (47%). Furthermore, a quarter of people identified a neurodevelopmental diagnosis.^b^

### DoI and associations with features of illness

The median DoI was 8.0 (IQR 4–15, *n* = 199), and had a significant correlation only with levels of current anxiety and a negative correlation with the age of onset of eating disorder behaviours (Table 2). There were 34 (17%) participants in the sample with DoI ≤3 years (median 2, IQR 1–3) or ES-AN, and 118 (59.3%) with DoI ≥7 years (median 14, IQR 10–20) or L-AN (as defined in this paper). DoI was unavailable for 9 people of the total sample (*n* = 208).

**Table 2:** Associations between duration of illness (DoI) and features of illness in 199 participants with persistent illness

Parameters	DoI (years)		
	*n*	Spearman *r*_s_	*P*
Body mass index (kg/m^2^)	143	−0.073	0.389
Eating Disorder Examination Questionnaire (Short)	199	0.007	0.919
Hospital Anxiety and Depression Scale (anxiety)	197	0.189	**0.008**
Hospital Anxiety and Depression Scale (depression)	197	0.053	0.461
Health-Related Quality of Life – Short Form 12 mental health component score	70	0	0.997
Mental health comorbidities (*n*)	64	0.077	0.544
Physical health comorbidities (*n*)	65	0.201	0.109
Age at eating disorder symptom onset (years)	158	−0.391	**<0.001**

Bold data denotes significant *P*-values.

As shown in Table 3, there were no significant differences between groups for BMI, levels of eating disorder symptoms (EDE-QS scores) or levels of depression or anxiety that approached levels of clinical severity. Mental HR-QoL was low (‘well below’ population means), but both this and physical health-related QoL were not significantly different between groups, despite there being significantly more mental and physical health comorbidities in the L-AN group.

**Table 3:** Features of people with persistent illness, comparing those in early stage (≤3 years) with longstanding (≥7 years) duration of illness (DoI)

Parameters	*n*		Mean (s.d.)		Statistic
	≤3 years	≥7 years	≤3 years	≥7 years	*t*-test *(P) d*
BMI (kg/m^2^)	26	81	20.2 (3.8)	20.8 (5.2)	−0.532 (0.596) 0.13
EDE-QS	34	118	17.5 (7.2)	18.4 (7.7)	−0.582 (0.561) 0.12
Age (years)	33	112	21 (4.95)	32.6 (13.2)	−4.96 **(<0.001)** 1.6
HADS-A	34	117	9.7 (3.7)	10.4 (4.6)	−0.834 (0.406) 0.16
HADS-D	34	117	9.6 (2.6)	9.5 (4.0)	0.252 (0.802) 0.03
			Median (IQR)		Mann−Whitney *U* (*P)*
HR-QoL (MCS)	7	56	41.24 (30.39–45.48)	34.2 (24.55–44.2)	159.0 (.433)
HR-QoL (PCS)	7	56	46.03 (38.33–51.29)	49.2 (40.6–58.9)	240.0 (.349)
Number of mental health comorbidities	6	52	2 (1-2.2)	3 (2-4)	248.00 **(0.024)**
Number of physical health comorbidities	7	53	2 (1-2)	3 (2-5)	285.00 **(0.02)**
DoI, years	34	113	2 (1–3)	14 (10–20)	4012.00 **(<0.001)**
Duration of untreated eating disorder, years	36	118	1 (0–2)	5 (1.5–10)	3151.00 **(<0.001)**
Total eating disorder care episodes	16	77	5 (3–6.75)	7 (5–10)	869.00 (**0.010)**
Number of in-patient/high-care admissions	34	118	1 (1–3)	3 (2–4)	854.50 **(0.013)**

BMI, body mass index; EDE-QS, Eating Disorder Examination Questionnaire (Short); HADS-A, Hospital Anxiety and Depression Scale (anxiety); HADS-D, Hospital Anxiety and Depression Scale (depression); IQR, interquartile range; HR-QoL, health-related quality of life; MCS, mental component score; PCS, physical component score.

Bold data denotes significant *P*-values.

### Exploring relationships between self-concept and identity (SCIM questionnaire) and illness experience

#### Quantitative findings: self-concept and identity

SCIM did not correlate with the overall duration of anorexia nervosa (Spearman’s correlation coefficient (*r*_**s**_) = −0.16, *P* = 0.919, *n* = 44). The total mean SCIM score of 90 for the L-AN group (*n* = 38, s.d. = 22.4) indicated an overall identity disturbance. Due to the scale being administered to the second cohort of participants in 2022, the sample size in the ES-AN group (*n* = 2) was too small for comparison with the L-AN group. Thus, we elected to analyse the qualitative survey responses for SCIM in the L-AN group using a thematic analysis. This analysis generated several themes, as shown in Supplementary 4. This table also includes exemplar quotes for each theme and its corresponding subthemes.

#### Qualitative findings (SCIM) informing quantitative tests

In reference to the SCIM thematic analysis (Supplementary 4), the first theme (disconnected self; ‘I wasn’t a real person’) describes points at which the participants identified themselves as most severely unwell (e.g. ‘at my lowest points’). They described experiences such as feeling ‘cloudy’ and less ‘real’ as a person, ‘numb’ and more of a ‘persona’, ‘broken’ and ‘empty’. There were also strong resonances with shame processes that interrupted with a coherent sense of self, with descriptions of feeling ‘vile’, ‘abhorrent’ as a person and ‘disgusting inside’. Shame was identified by some as a driver for self-destruction: ‘that shame I felt to my core causing me to starve to death’. This qualitative finding informed the statistical correlation between eating disorder symptom severity (EDE-QS score) and disrupted sense of self (SCIM), which was found to be significant (*r*_s_ = 0.427, *P* = 0.003).

#### Qualitative findings (SCIM) thematic analysis

The second of three themes (self and recovery processes) encompassed two subthemes: making sense of self and anorexia nervosa (theme 2a) and disentangling self, healing shame (theme 2b) (see Supplementary 4). For many participants, developing and strengthening their sense of themselves, as well as finding value in themselves as a person (healing shame), was reported as a key recovery process: ‘Recovery itself is an incredible way of being forced to value yourself. I don’t know that I would’ve faced myself otherwise. It’s a gift in a way’. Recovery was not finite and did not involve recovering to a pre-eating disorder version of self or dichotomously rejecting the eating disorder, for example:

‘The eating disorder has ended up becoming a very prominent part of my life, even now from a recovery-oriented perspective and helping others who are struggling… both are equally me, and for the majority of my life it’s felt that they were opposites. I am now trying to bridge those gaps which is why I feel less “broken”’. (Participant 36)

Rather, the participants’ accounts reflected a sense that they could be changed and transformed in self-enhancing ways by the eating disorder experience, by disentangling it from themselves and selectively integrating aspects of the experience, including their survival. One participant remarked: ‘I have learnt how resilient and determined I can be’. For some people, this process involved making sense of their place in the world. It included, for some who had experienced childhood trauma, an understanding of how these experiences were key in recruiting them into shame – for example:

‘I strongly believe trauma plays a driving factor in most eating disorders, and these questions [sense of self] are important because trauma, particularly in childhood, often leads to a lack of sense of self or identity’. (Participant 77)

Likewise, the impact of experiences such as dissociation and autism spectrum was described by some as complicating the development of a ‘solid sense of self’ (Supplementary 4). Within these core themes each emphasised the complexity of the anorexia nervosa experience, and thus recovery was noted as requiring time (theme 3a: It took time). The combination of a need to focus on questions of identity, along with the understanding that this takes time, was cited as one of the key challenges to participants’ engagement with treatments. Some of these experiences included the sense of not being treated as a person, questions of ‘Who I am’ not being addressed in treatment and, for some, experiences described as treatment trauma that compounded previous, often unaddressed, traumatic life experiences (see Supplementary 4, theme 3b: treatment harm and limitations).

### Understanding people’s treatment experiences

#### Types of individual therapy treatments experienced

Based on self-reported individual eating disorder treatment types, nearly all people reported experiencing CBT, and dialectical behaviour therapy (DBT) was also commonly experienced (Table 4). The least frequently reported treatment was specialist supportive clinical management (SSCM), and no one in the ES-AN group reported receiving SSCM. There were no significant differences in the proportions of people with L-AN and ES-AN who reported receiving CBT, DBT or mindfulness-based therapy (*χ* ^2^ with Yates correction 0.31, 0.81, 0.03, respectively; all *P* > 0.05). No statistical comparisons were undertaken for other psychotherapies, due to very low cell sizes.

**Table 4:** Comparing the types of individual therapy reported by those at early stage (≤3 years) with those of longstanding (≥7 years) duration of illness

Parameters	All (*n* = 184), *n* (%)	AN ≤3 years (*n* = 30), *n* (%)	AN ≥7 years (*n* = 106), *n* (%)
Cognitive–behavioural therapy	169 (91.8)	29 (96.7)	97 (91.5)
Dialectical behaviour therapy	112 (60.9)	15 (50)	65 (61.3)
Mindfulness-based therapy	112 (60.9)	19 (63.3)	63 (59.4)
Psychotherapy	70 (38.0)	5 (16.7)	50 (47.2)
Acceptance and commitment therapy	63 (34.2)	7 (23.3)	40 (37.7)
Behaviour therapy	46 (25.0)	4 (13.3)	27 (25.5)
Motivational interviewing	29 (15.8)	3 (10)	23 (21.7)
Narrative therapy	20 (10.9)	3 (10)	16 (15.1)
Psychodynamic psychotherapy	23 (12.5)	2 (6.7)	18 (17.0)
Interpersonal psychotherapy	9 (4.9)	0 (0)	7 (6.6)
MANTRA	7 (3.8)	0 (0)	6 (5.7)
Specialist supportive clinical management	2 (1.1)	0 (0)	2 (1.9)
Unsure	13 (7.1)	3 (9.7)	9 (8.5)
Other unspecified	11 (6.0)	1 (3.3)	9 (8.5)

AN, anorexia nervosa; MANTRA, Maudsley Anorexia Nervosa Treatment for Adults.

#### Least and most helpful treatment types

Utilising participant descriptions of the least and most helpful treatments, treatments were categorised by type. Of the least helpful treatment (LHT) experiences, around half were reported to be in an in-patient setting, most often in a general mental health unit. Some encompassed a behaviour modification philosophy, for example:

‘Bed rest and deprivation and withdrawal of basic human rights. I was in a room on my own as an 11-year-old with no books or TV. I was not allowed contact with my family. I had to use a bed pan and was not allowed to wash or brush my teeth until I gained weight. I was force-fed until I was sick which made me ashamed. Being treated like a bad child. Forced [naso-gastric] tubing’. (Participant 6)

‘In-patient hospital stay was structured in a punitive and coercive way. Though I went voluntarily, I did so because I was scared I was going to die… I had to give up all of my freedoms and was being controlled by others 24/7. If I couldn’t finish a meal, I would have things taken away. I would not be allowed to see friends, or have leave from the ward. I missed my grandmother’s funeral, my cousin’s wedding,… and many other things. All for absolutely no reason… we were barred from having normal lives’. (Participant 10)

These extracts powerfully portray the question for treatment providers of how to respond to a person who may be close to death without also traumatising them.

The most helpful treatment (MHT) was out-patient treatment (61%), where people reported a preference for an individualised, non-generic form of individual psychological therapy (44%) and/or within a multidisciplinary out-patient team approach (20%). This could provide the required choices and, at the same time, freedom around change. Although the second most frequently reported LHT was CBT (36%), the following extracts highlight the importance of considering the person’s preference, with each of these participants citing CBT as either their MHT or LHT, overall.

‘Inflexible, focused on weight and body (ironically), shaming when I didn’t complete homework, NOT a therapy, the clinician did not develop rapport, felt like a lab rat. Hated this experience. Made me feel like even more of a failure’. (Participant 135)

‘It was helpful that I started chipping away at my cognitive distortions [with CBT] and the never-ending list of rules I had formed. Looking back, it blows me away how much I believed to be true. I think it was also so very helpful to look at the relational aspect with IPT [interpersonal therapy]’. (Participant 125)

This also emphasises the need for personalised treatment, because what is helpful for one person may not be beneficial for all.

#### Qualitative analyses and understanding treatment experiences

A second thematic analysis, of open-ended treatment experience questions, is presented visually in Fig. 1. The voices of 175 individuals with experiences of treatment for anorexia nervosa are represented in the analysis and two major themes are presented, grouped as the ‘what’ and ‘how’ of treatments (Fig. 1).

The LHT experience was significantly less helpful overall for the L-AN group (Tables 5 and 6). For treatment to be well received, the qualitative analysis highlighted two themes, (a) the ‘how’ and (b) ‘what’ of treatment, which encompassed a synergy of several key elements (Fig. 1). How a treatment is delivered (Fig. 1) included: theme a(i),‘therapeutic philosophy’, theme a(ii), ‘individualised and personalised care’ and theme a(iii), ‘autonomy and choice’, interacting with ‘what’ (theme b) is delivered. The ‘what’ components included b(i) systems of care, b(ii) a banquet of options and b(iii) strengthened self.

**Table 5:** Associations between duration of illness and treatment experiences in 199 participants with persistent illness

Treatment factors	Median (IQR) *n*	Spearman *r*_s_	*P*
Total eating disorder care episodes	6 (5–9) 122	0.207	**0.024**
Number of in-patient admissions	2 (1–4) 123	0.309	**<0.001**
Duration of untreated eating disorder, years	2 (1–6) 198	0.501	**<0.001**
Extent to which a person felt understood by the therapist (MHT)	42.5 (24.75 to 50.0) 190	0.145	**0.046**
Extent to which the treatment approach gave freedom to make own choices around change (MHT)	33 (13.25 to 46) 191	0.149	**0.049**
Extent to which the treatment approach considered personal treatment preferences (LHT)	−44 (−50 to −24) 191	−0.234	**0.001**
Extent to which the treatment approach gave freedom to make own choices around change (LHT)	−44 (−50 to −12.75) 191	−0.185	**0.010**
Extent to which the therapist instilled hope for recovery (LHT)	−26.5 (−50 to 23.25) 189	−0.197	**0.007**
Overall helpfulness of LHT	−40.0 (−50 to −18) 190	−0.174	**0.016**

IQR, interquartile range; MHT, most helpful treatment; LHT, least helpful treatment.

Bold data denotes significant *P*-values.

**Table 6:** Participants’ perceptions of the most and least helpful eating disorder treatment experiences, comparing those of short (≤3 years) with longer (≥7 years) duration of anorexia nervosa

Perceptions	*n*		Median (IQR)		Mann–Whitney *U* (*P)*
	≤3 years	≥7 years	≤3 years	≥7 years	
Most helpful treatment ratings^a^					
Extent to which the treatment approach considered personal treatment preferences	33	110	24 (3 to 49)	36.5 (20.5 to 47)	2189.50 (0.087)
Extent to which the treatment approach gave freedom to make own choices around change	33	110	21 (–9.5 to 38.5)	36 (21 to 49)	2374.50 (**0.009**)
Extent to which the therapist instilled hope for recovery	33	110	39 (15 to 50)	42.5 (21 to 50)	1901.00 (0.670)
Extent to which a person felt understood by the therapist	33	110	30 (2.5 to 50)	43 (28.75 to 50)	2216.50 (**0.047**)
Extent to which the therapist addressed personal concerns	33	109	32 (13 to 50)	43 (27 to 50)	2187.50 (**0.055**)
Overall helpfulness of this treatment	33	110	28 (17 to 50)	41 (20 to 50)	1983.50 (0.461)
Least helpful treatment ratings					
Extent to which the treatment approach considered personal treatment preferences	33	108	−24 (−44 to 0)	−50 (−50 to −29)	1074.50 **(<0.001)**
Extent to which the treatment approach gave freedom to make own choices around change	33	108	−12 (−50 to 16.5)	−49.5 (−50 to −25.5)	1227.50 **(0.003)**
Extent to which the therapist instilled hope for recovery	33	108	18 (−40 to 38)	−39.5 (−50 to 16.5)	1174.00 **(0.002)**
Extent to which a person felt understood by the therapist	33	108	−28 (−50 to −19.5)	−50 (−50 to −21.25)	1335.50 **(0.012)**
Extent to which the therapist addressed personal concerns	33	108	−18 (−43 to 19.5)	−48 (−50 to −11.25)	1215.00 **(0.003)**
Overall helpfulness of this treatment	33	108	−25 (−46.5 to 5.5)	−46 (−50 to −21.25)	1244.00 (**0.005**)
	*n*		*n* (%)		*χ* ^2^; *P*
Other treatment features					
Underwent in-patient treatment (hospital)	33	113	18 (54.5)	85 (75.2)	5.255; **0.022** (d.f. = 1)
Least helpful treatment was voluntary	32	112	22 (68.75)	73 (65.2)	0.141; 0.707 (d.f. = 1)
Most helpful treatment was voluntary	33	112	26 (79)	96 (86)	0.916; 0.338 (d.f. = 1)

IQR, interquartile range.

a. Most and least helpful treatment experiences (according to Treatment Experiences Sliding Scale Rater) were ranked from –50 (most negative) to 50 (most positive).

Bold data denotes significant *P*-values.

#### Exploration of perceived treatment experiences and associations with DoI

As shown in Table 3, compared with those with ES-AN, individuals with L-AN had a significantly longer DUED and more episodes of care. DoI had significant correlations with the number of eating disorder care episodes and hospitalisations, DUED and negative experiences, particularly, but not only, with their perceived most unhelpful treatment (Table 5).

#### DUED

The level of DUED was five times higher for the L-AN group, and the difference was highly significant (Table 3). Despite a longer delay in receiving treatment, people with L-AN experienced significantly more episodes of care for their eating disorder than the ES-AN group. Several participants commented on the importance of timely treatment, and reflected that systemic and personal barriers could contribute to treatment delays:

‘I believe things would not have gotten so out of control for me if I had been allowed to get treatment right away’. (Participant 5)

‘I would encourage anyone who is able to pursue recovery/treatment as quickly as possible after being diagnosed. I feel like I had so much more hope and motivation that things could change in the first few years, but it’s now 15+ years since I was first diagnosed and I’m starting to feel the physical effects as well as the psychological. Again, it’s my own fault, but I think attacking the fear with as much gusto as possible early on is so important’. (Participant 72)

Specific treatment inputs, distilled through thematic analysis (Fig. 1), emphasised the need for a ‘system of healthcare’ (theme b(i)) to identify the eating disorder and deliver the required level of care. One participant stated, ‘Back in the 1970s in the UK, there wasn’t any treatment’. Eating disorder behaviours could also be normalised, minimised or undetected by healthcare – for example:

‘No one ever asked me [about an eating disorder]. I was panting on the side of the road when I was running, so I got admitted to a big hospital with a heart rate in the thirties and they were like I don’t know you have a very slow heart rate. It’s not safe, but we can’t figure out why. No one ever asked me, I was never weighed, was fainting every time I stood up… they did a lumbar puncture, and nobody was asking… I was super unwell. 30+ years with an eating disorder. I was seen as fit [healthy]’. (Participant 17)

#### Treatment intensity

People with L-AN were significantly more likely to have undertaken more intensive care (e.g. in-patient) treatment (Table 6). There was a significant negative correlation between the total number of episodes of care and the helpfulness ratings of LHT (*r*_s_ = −0.233, *P* = 0.012). There was no correlation between the number of episodes of care and the overall helpfulness rating of MHT (*r*_s_ = 0.037, *P* = 0.687. However, there was a significant negative correlation between DoI and helpfulness ratings of LHT (*r*_s_ = −0.174, *P* = 0.016), and negative ratings of experiences of LHT were higher in those with L-AN (see Ratings of treatment experiences section below). There was no correlation between DoI and overall ratings of the helpfulness of MHT (*r*_s_ = 0.004, *P* = 0.961).

Thus, people with longer DoI were more likely to report poorer treatment experiences, and more episodes of treatment with greater intensity and delay between first behaviours and first treatment, than those with shorter duration.

#### Ratings of treatment experiences

##### Features of LHT

People with L-AN reported significantly lower ratings for the following perceptions of their LHT, as quantified by TESSR ratings: ‘treatment taking into account personal preferences’, ‘giving freedom around change’, ‘feeling understood by therapist’, ‘instilling hope for recovery’ and ‘overall helpfulness’ (Table 6). In regard to MHT, freedom around change, as well as therapist factors such as feeling understood and addressing concerns, were reported as having significantly lower ratings for the L-AN group (Table 6).

##### TESSR ratings for ‘instilling hope for recovery’ and the ‘therapeutic relationship’ explained by the thematic analysis (the ‘how’ of treatment)

The MHT was highly rated for instilling hope in both groups (Table 6) and, in LHT, hope was significantly lacking for the L-AN group (Tables 5 and 6). Hope and the therapeutic relationship were highlighted in the qualitative analysis as being intrinsically linked. The therapeutic philosophy (Fig. 1; theme a(i)) of helpful treatments linked to recovery emphasised features such as ‘listening’, ‘connection’, ‘trust’, ‘safety’, ‘compassion’ and ‘respect’, allowing a person to be ‘challenged towards growth’. At the same time, their suffering was also acknowledged and validated. This assisted people in feeling ‘loved back to life and not giving up and being validated… hold the hope for me when I wasn’t able to hold it for myself’ (Fig. 1). Thus, the therapeutic relationship was described by some participants as a foundation for hope and, in the absence of this, a range of detrimental effects were described, for example:

‘[…] treatment in public both out-patient, in-patient, and medical; It made me worse, left me with trauma, took away most of my hope, made me feel less than, not listened to. Was told “play the game”, no support, no care, you’re not a human, you’re a number. It makes you feel worthless’. (Participant 29)

Treatment experiences that did not prioritise these therapeutic factors (feeling understood and addressing personal concerns) were perceived to undermine a sense of self-worth and connection to hope for recovery.

In reference to Fig. 1, participants consistently expressed a preference for treatments that were individualised and personalised (theme a(ii)), such that the person was not ‘just treated like a number’ or treated ‘based on numbers’ (i.e. weight stigma and weight-centric care) and, critically, progressed at their own pace. For example:

‘[…] built a stable foundation by working through the trauma that made me this way and built up on that and challenged me in my own pace, while slowly getting me out of my comfort zone’. (Participant 166)

##### TESSR ratings for ‘giving freedom around change’

The qualitative analysis expanded on the findings that people with L-AN perceived greater freedom to make their own choices regarding change in their MHT, and less freedom to make their own choices regarding change in their LHT (Table 6). Autonomy and choice (Fig. 1, theme a(iii)) were described as a person feeling that their own needs were heard and respected, as opposed to being ‘restrained’ and ‘threatened’ into change that they did not yet feel able to support within themselves (see examples of quotes in Fig. 1).

### Freedom: involuntary treatment

The proportion of people who reported receiving their LHT involuntarily (Table 6) (71 of 198, 36%) was greater than that who reported their most helpful treatment as involuntary (32 of 199, 16%) (*χ* ^2^ = 22.03, d.f. = 1, *P* < 0.001). The proportions of involuntary treatment were comparable between ES-AN and L-AN groups for both LHT and MHT. The treatment experience rated by participants as least helpful was involuntary for roughly a third of people in the L-AN group (*n* = 112, 35%) and a fifth in the ES-AN group (*n* = 33, 21%) (*χ* ^2^ = 0.141, d.f. = 1, *P* = 0.707). The treatment experience rated by participants as most helpful was involuntary for 21% of ES-AN participants (*n* = 33) and 14% of L-AN participants (*n* = 112) (*χ* ^2^ = 0.916, d.f. = 1, *P* = 0.338). Contrary to our hypothesis, those with ES-AN were just as likely to have experienced their least and most helpful treatments involuntarily. Participant interpretation of the term ‘involuntary’ ranged from ‘parental insistence’ to treatment ordered under an Australian Mental Health Act. Participants remarked that it was surprising to them that, despite being voluntary patients, they were denied input into their own care.

### Treatment systems and components: the treatment ‘what’

Equally important to people in regard to how a treatment is delivered was the presence a system of care (theme b(i)) that could deliver on appropriate levels of care such that treatment was personalised and individualised to the person who could ‘step up to a higher level of care’ and be supported within a system or ‘a whole treatment team’ (Fig. 1). Participants remarked that there was no single ‘fix-all’ method. Instead, people described needing many different types of treatment ‘banquet of options: all of it’ (theme b (ii)) and at different stages of recovery, often more than once and over a long period. This is consistent with quantitative findings that people with L-AN had experienced significantly more episodes of treatment (Table 3) and that most individuals had undergone extensive treatment. Thus, when an eating disorder diagnosis was affirmed there was at times no appropriate help, and this impacted treatment delay and protracted recovery.

#### TESSR ratings for ‘overall helpfulness’: strengthening the person within the illness

Underlying the more helpful treatment experiences, and notably absent from those that were least helpful, was how a treatment could support a person towards a ‘strengthened self’ (theme b(iii)). This permeated many facets of treatment, including physical nourishment, as well as ‘reconnecting with body cues and needs’ and having their needs listened to and respected via interpersonal trust, affirming them as worthy. The extent to which a person felt understood by their therapist or, in other words, seen for who they are, was rated significantly lower in the LHT experience for those in the L-AN group (Table 4 and 6). With a stronger self-connection (i.e. a strengthened self), the person can engage in healing processes, as described by two participants as follows: ‘build a life outside of an eating disorder’ as opposed to ‘reinforcing that the eating disorder was a major part of my life and identity’.

Conversely, an impoverishment of self could be perpetuated by treatments that were perceived as taking control from them (see freedom and personal preferences ratings in Tables 5 and 6) and made them feel irrelevant: for example, ‘It [treatment] made me feel pointless because they hardly acknowledged me. It was like people were talking about controlling my life right in front of me’. Feeling disregarded as a person was experienced when treatment focused predominantly on (a) weight and eating disorder symptom improvement rather than the person (for example: ‘I don’t think it focused on long-term recovery. The metric was getting my weight up and I didn’t feel supported once this ended’); and (b) through applying a non-personalised approach (for example, a ‘one-size-fits-all standard process’). Some participants described this as minimising (for example, ‘left me voiceless’) and eroding trust in themselves (for example, ‘took me away from trusting myself’). Notably, these descriptions were counter to those described when participants were engaging in treatment processes that contributed to a ‘strengthened self’ (Fig. 1, theme b(ii)), which was perceived as a key recovery process for a number of participants.

Overwhelmingly, treatment was described as falling short for many participants, particularly in failing to provide a holistic focus on them as individuals and systemic limitations. Scores of identity/self-disturbance, and participants’ emphasis that treatments often did not address ‘self’ processes adequately, appeared in many accounts as exacerbating their vulnerable sense of self. For example, one participant stated: ‘I felt like there was something wrong with me, and if I were a proper/complete person, it [treatment] would have been a better fit’ (Supplementary 4, theme 3b: treatment harm and limitations). A clear picture emerged in which treatment had failed to adequately address this core feature of anorexia nervosa illness for those experiencing L-AN, and arose in both analyses (Fig. 1 and Supplementary 4). Therefore, participant accounts indicated that a persistent illness was at least partially iatrogenic.

## Discussion

The present study, to our knowledge, is the first to test the formal definition of SE-AN proposed by Hay and Touyz in 2018.^29^ The study aimed to describe a community sample of treatment-seeking individuals and test the putative features of anorexia nervosa between those of shorter (≤3 years) and longer (≥7 years) illness duration, modelled on delineations adopted by Ambwani and colleagues^38^ and others. Descriptive terminology of ES-AN (≤3 years) or L-AN (≥7 years) was used. Between-group comparisons and correlations aimed to refine SE-AN nosology, enhance conceptualisations of anorexia nervosa and advance treatments.

A cross-sectional community sample of treatment-seeking individuals identified 208 participants who were reportedly symptomatic for anorexia nervosa across 6 countries. Their illness experience was characterised as long (mean of 8 years), and occurred with multiple psychiatric comorbidities and/or aversive life experiences. They reported many eating disorder treatments (most commonly CBT) and additional treatment for comorbid psychiatric diagnoses. A short (≤3 years) illness experience had occurred for the minority (17%). This is unsurprising given that recovery (i.e. symptom remission) in the first 9 years of illness is uncommon.^89^ In summary, the participants in the study group can be described as having been unwell for a long time, with complex presentations and receiving extensive treatment. Consistent with Ambwani and colleagues,^38^ the L-AN group was more likely to require intensive ‘step-up’ treatment episodes than those with shorter duration; however, we did not observe the same differences in other outcome measures. Our L-AN group had experienced significantly more treatment episodes and were older than those with ES-AN and, in contrast to Ambwani et al’s sample, were a community, self-report sample of people. There were no exclusions made based on BMI (whereas a BMI of <18.5 kg/m^2^ was required for inclusion in the former sample^33^) or ‘distress’ scores for depression and anxiety. Therefore, there may be differences in treatment trajectories between clinical and community groups, whereby longstanding experiences in the community may be distinct from specialised community eating disorder services. This is particularly the case for those with a higher BMI, whose longstanding illness may not present to, or be accepted by, tertiary clinics. Weight stigma emerged in the qualitative analysis as a barrier to treatment access, which supports this explanation and provides further evidence for discussions about the harms of the ‘atypical’ weight-based diagnostic construct.^34–36^

When comparing ES-AN with L-AN using correlations and between-group comparisons, no differences were found in BMI, and both groups exhibited clinically elevated markers of anorexia nervosa severity, as measured by eating disorder E-QS, HADS (anxiety), mental HR-QoL and SCIM. Except for a trend towards higher levels of anxiety for L-AN, none of the severity indices were significantly different. Of note, the correlation coefficient for duration versus BMI was almost linear on the scatter plot up to the first 20 years. Therefore, defining an L-AN state based on the severity of low weight was not supported. Similarly, severity, as defined by EDE-QS, did not relate to duration. Irrespective of the spectrum of duration, all anorexia nervosa may be severe when considering these factors.

Although people with L-AN reported a significantly greater number of physical health problems, this did not translate to an overall perception of a lower physical HR-QoL. This conflicts with other research that has found that a long DoI is associated with a lower quality of life, and may speak to personality factors of people in the present sample, such as stoicism, resilience or denial of the impact of anorexia nervosa, as noted by others.^30^ It is also possible that people make adaptations to their physical health and comorbidities over time, and further qualitative research could explore the experiences of the physical impacts of anorexia nervosa, acknowledging that they are manifest.

Building on previous research as part of this overall study (i.e. phase 2; see Supplementary 1 ^28,40,69^)and elsewhere,^90^ identity disturbance, including a loss of a sense of self, was hypothesised to be part of the L-AN experience. The qualitative strand of the present research highlighted the connection between identity disturbance, severity of illness and recovery. Additionally, participants’ SCIM scores indicated comparatively higher disturbance than in other clinical groups^91^ and matched with another anorexia nervosa group.^92^ However, although identity disturbance correlated with higher symptom severity (as measured by EDE-QS), it was unrelated to duration. As such, identity disturbance as measured by SCIM appears to be a severity feature of anorexia nervosa irrespective of DoI. An eating disorder-specific identity tool might also be required with attention to developmental milestones, autism spectrum disorder, shame and dissociative processes as complicating the sense of self. Our hypothesis was therefore only partially supported by the present findings (i.e. related to severity but not duration). Therefore, our earlier suggestion, to consider identity disturbance within SE-AN nosology,^69^ is not supported because it appears to be an overlooked feature of anorexia nervosa more broadly.

Overall, these results, combined with the findings reported on treatment experiences, suggest that, at any time point, anorexia nervosa is severe; however, if it is enduring (longstanding) this may be more so due to treatment-related factors. These encompassed the following: long DUED, high number of treatment episodes, higher treatment unhelpfulness ratings and overlooked features of illness in current treatments.

The average DUED in people with L-AN in this study was 5 times higher than the 1 year required for ES-AN to commence treatment. A systematic review of 14 studies^33^ found the average DUED for anorexia nervosa to be 29.9 months. Comparatively, in our study, the L-AN group reported double the length of this average DUED and, for the ES-AN group, it was reported as half. Research suggests that early interventions yield improved outcomes for individuals,^12,14,57^ and the present study supports these findings. The L-AN group was significantly older than the ES-AN group, which could indicate that timely access to treatment has improved; however, we cannot comment on the specific year(s) when treatments were undertaken.

The significant finding – that the longer the DoI without treatment (i.e. DUED) the poorer the outcome for people – corroborates findings by other researchers. For example, a study by Fukutomi and colleagues^12^ demonstrated that, while the frequency of service utilisation was the same between a ‘rapid’ intervention group and ‘treatment as usual’ group, a lower intensity of treatment was required and weight recovery was better achieved in the former group.

Participants in this study had undergone a significant number of treatments, and those with a longstanding experience had a significantly higher total number of treatments than those with shorter durations. When people persisted with treatment, they eventually found a treatment that was more acceptable to them. This poses a challenge to the pejorative term ‘treatment resistance’.^93^ Participant accounts indicated that they had engaged repeatedly with treatment, and these accounts were inconsistent with the understanding that they had resisted the treatment, even though they found it highly unsatisfactory at times. Helpful treatment is beneficial at any time, and unhelpful treatment becomes less effective over time, especially as the number of treatments increases. This suggests that persisting with unhelpful treatment can tip into a harmful experience and loss of hope. However, we cannot comment on the timing within the treatment trajectory when people experienced their LHT and how this impacted their perceptions of future treatments; further studies could investigate this topic.

Treatment preferences had the highest correlation with duration, pointing to a need to revise treatments, including the consideration of ‘how’ treatment is delivered and the focus of treatment (i.e. ‘what’), as summarised in Fig. 1, particularly if the person identifies that treatment is not helpful for them. The impact of meeting a person’s treatment needs early in the trajectory could reduce the number and duration of treatments, but further longitudinal research is required to confirm this. Although it is unknown whether the treatments people received were evidence-based, and we cannot comment on the quality of delivery, participant responses were very detailed. They used terminology indicative of high treatment literacy and our assumption, based on qualitative findings, is that some of the treatments experienced had not met a person’s needs or engaged them. Multiple treatments are necessary for L-AN, but these must also meet people’s needs and preferences and be readily available within a comprehensive system of care (see Fig. 1). It is unknown what impact meeting a person’s treatment needs early on in their treatment encounters would have on the total number of treatments. The finding that treatments have not met individual needs and preferences is echoed throughout the qualitative literature on anorexia nervosa treatment experiences elsewhere,^41,94,95^ and this study represents a quantification of these findings.

In terms of the types of treatment it was notable that SSCM, which has shown particularly high engagement and encouraging outcomes (QoL and eating disorder symptoms) in the only randomised control trial conducted for L-AN,^26^ was the least likely to have been reported as received. This suggests that there are efficacious treatment options^96^ still to be explored for people with L-AN, despite an overall high number of treatment episodes. This offers hope to those with L-AN and their clinicians to continue to seek treatment options in line with their needs and preferences, and cautions against labelling people as ‘severe and enduring’ or ‘treatment-resistant’. Participants rated most frequently non-specific in-patient treatment and out-patient CBT as their LHT. This may be because they are perceived to have less scope to meet the most helpful features described in Fig. 1 (personalised, individualised and autonomous) when CBT is delivered in a strictly manualised manner and in-patient treatment without sufficient autonomy. It may also be reflective of the needs of this sample, being a group of people with anorexia nervosa with high comorbidity and co-occurring adverse life events, such as childhood neglect and abuse.^97^ A 20-year longitudinal study^4^ reported a comparatively lower anorexia nervosa recovery rate of 40% at 20 years (compared with 50–80% in other studies^3,98,99^). In accounting for this discrepancy, they reported that ‘All of our patients were treated with a considerable amount of intensive in-patient CBT-E [cognitive–behavioural therapy enhanced]’^4^) and considered this indicative of greater severity. As an alternative hypothesis in the context of the present research, perhaps the repeated delivery of this (in-patient and CBT), and/or any type of treatment that is not working, is also a factor in the comparatively poor outcomes discovered.

There is compelling evidence^40,91,92,99^ to suggest that self and identity disturbances are both predisposing and perpetuating factors in anorexia nervosa. Identity disturbance is an index of severity, a feature of anorexia nervosa illness (irrespective of duration) and a marker of self-reported recovery. Strengthening a sense of ‘self’ was revealed as key to recovery processes in anorexia nervosa, in both qualitative aspects of this study (Fig. 1 and Supplementary 4) and research by others.^101^ In other words, a long illness does not necessarily mean greater disturbance to a person’s identity. A greater focus on a sense of self and identity as a key feature of treatment is warranted, and this should occur across the spectrum of illness, delivered in a way that fosters a person’s agency, self-efficacy and self-development. Furthermore, how treatments might be adapted to consider shame processes (i.e. shame-sensitive) is under-recognised and incomplete in eating disorder treatment.^102–105^

Most first-line treatments are based on cognitive–interpersonal (Maudsley Model of Anorexia Nervosa Treatment for Adults (MANTRA^106^) and cognitive–behavioural models.^107^ While there has been some focus on a sense of self and identity in selected forms of CBT, such as that used in the SE-AN clinical trial^108^ and in MANTRA,^109^ this has not been comprehensively considered in treatments more broadly. When this is addressed, it is typically contingent on weight restoration. But what if weight restoration is not achieved? But what if weight restoration is not achieved? There is a need for greater flexibility in treatments to address what the person is ready, willing and able to address, including questions of identity, while balancing medical risk. Furthermore, tailoring treatments to be responsive to what is now known about intersections of trauma, neurodiversity and gender and given the high levels of sensitivity consistently reported by those with anorexia nervosa,^28,41,45^ this implies that the manner in which treatments are delivered is probably as important as the choice of treatments. As it stands, it is well established that few people engage in eating disorder treatments,^53^ for reasons encompassing systemic healthcare issues,^110^ lack of detection of eating disorders^111^ and individual barriers including shame, perceived unworthiness^40,80^ and stigma.^112^

### Implications for L-AN nosology and future directions

There is mixed evidence for a distinct L-AN diagnostic category in this study. The prefix of ‘severe’ in the proposed definition of ‘severe and enduring’ is not appropriate: anorexia nervosa remained as severe, despite treatment, at both early and later stages in the present study. Typically, anorexia nervosa persists for 9 years for at least 60% of people according to medicalised recovery definitions,^3^ and in this study was found to be ongoing at a median of 8 years (IQR 4–15) despite a median of 6 episodes of care (IQR 5–9) in the overall sample (*n* = 199) and 14 years (IQR 10–20), with a median of 7 episodes of care (IQR 5–10) in the L-AN group. Collectively, the lack of empirical support for ‘severity’, uncertainty about broader recovery definitions,^28,113^ legacy of treatment inadequacy (availability and efficacy), low treatment satisfaction, high DUED and reported harms (stigma and loss of hope) of the SE-AN diagnostic label indicate that efforts should be channelled towards reconceptualising L-AN, revising treatments and assisting people towards treatment that meets their needs, without delay. In terms of duration of illness, there is unlikely to be a categorical, discrete category for duration and a dimensional diagnostic approach to nosology may be indicated,^114^ which would include consideration of a person’s treatment history. Furthermore, some authors on the topic of labelling and defining longstanding anorexia nervosa,^7,115^ but not all,^22,116^ have advocated for excluding categorical BMI cut-offs in L-AN nosology. The present research highlights the limitations and harm of BMI in the diagnosis of anorexia nervosa, because the sample in this study had a low to normal BMI yet still experienced functional impairment. This probably represents a clinical reality that not all people with anorexia nervosa and L-AN are severely underweight in terms of BMI, and further research is warranted concerning this aspect of nosology.

### Clinical implications

The practical factors (identification, access and lack of appropriate treatment) described as influencing a long DUED speak to the need to prioritise early intervention and ongoing care. This is consistent with other literature that implicates ‘treatment inadequacy’ and ‘systemic inequities’ as factors in illness persistence.^18,21,61,68,117^ There is also a risk that those with longer duration and higher age continue to be disadvantaged by a lack of access to rapid intervention services^19^ as an ongoing legacy of treatment inadequacy. Furthermore, the relationship between long DUED and neuroprogressive, neurodegenerative factors warrants further study.^118–120^ Nonetheless, there exists a substantial proportion of people with anorexia nervosa who would benefit from treatment innovation and care delivered in line with their treatment preferences, and this group cannot be ignored.

The current study indicates that a dimensional, rather than a categorical, approach, characterised by a need for multiple treatment episodes, may be more appropriate. That is, a personalised formulation should inform an individualised and tailored approach to treatment^121^ that draws on the wisdom of the experiencing person as to what they need, when and how. Further research should also explore how the neurobiological underpinnings can be applied in such an approach.^122^ Additionally, consideration needs to be given to reducing the long DUED and building in an expectation that ongoing treatment is probably indicated, albeit this takes time. Such an approach can foster hope within systems of care for providers, people with anorexia nervosa and their families.

### Study strengths and limitations

This research should be interpreted in light of several limitations. Based on the depths of responses, the sample represents people with high treatment literacy. The extent to which this shaped and impacted on the results is unknown. The sample could also be representative of those who feel they have a story to tell (i.e. have had particularly aversive treatment experiences) and may not generalise to others. The impact of untreated conditions on a person’s trajectory is unknown, and further studies could investigate this further. The total number of care episodes is probably underestimated in this research, as we did not specify the number of day-patient treatment experiences. Consistent with the iterative mixed-methods approach, some measures were not collected during the first administration of the survey and/or were reached by only a selected number of respondents in the second survey. This limited sample size means that the results were underpowered for some between-group comparisons, because only a few respondents had a short illness duration. The underpowered samples, e.g. self-reported comorbid diagnosis and psychiatric experiences, represent an area for future research. In addition, the results cannot extend in application to seekers of non-treatment (who represent the majority of those with eating disorders), and their illness trajectories remain unknown. Generalisability could also be enhanced through further longitudinal research, extending beyond the present cross-sectional and self-report design.

Some of the instruments used to measure severity (e.g. HADS for anxiety and depression) focused on acute changes in mood within the past week. Many participants reported a chronic experience of depression and anxiety; they commented that it was no better or worse in the weeks leading up to the survey. They felt this could have underestimated the severity of their depression and anxiety, and this may account for the lower than predicted distress scores.

Additionally, the sample remained relatively homogeneous in terms of diversity. We cannot comment on the total number of involuntary care episodes and the overall likelihood of having received involuntary treatment, so this hypothesis is only partially answered.

A strength was that respondents were highly engaged in the survey, providing detailed responses to open-ended questions. In addition to a commitment to completing a long and detailed survey, more than half of participants expressed a willingness to be interviewed for future research. The mixed-methods design is a strength of this research: it has highlighted the importance of qualitative research in capturing nuance, thereby enabling a more comprehensive expansion of findings and the generation of hypotheses.

In conclusion, the overall finding of this mixed-methods research is that L-AN may be more closely related to the total number and quality of treatment experiences, rather than to severity specifiers. Based on this research, the longstanding aspect may be iatrogenic for some. While the message is that people can improve with evidence-based treatments that are tailored to their needs and preferences, irrespective of the length or severity of their eating disorder, the importance of holding hope for people with longstanding anorexia nervosa is underscored. Conversely, repeating unhelpful treatments may undermine hope and unnecessarily protract the illness. Regardless of the utility of distinguishing between longstanding and ‘severe’ cases, outcomes for anorexia nervosa are unacceptably poor. Overall, there needs to be a greater focus on reconceptualising the condition and its treatments through longitudinal studies and further research into under-researched treatment interventions.

## Supporting information

Kiely et al. supplementary material 1 Kiely et al. supplementary material

Kiely et al. supplementary material 2 Kiely et al. supplementary material

Kiely et al. supplementary material 3 Kiely et al. supplementary material

Kiely et al. supplementary material 4 Kiely et al. supplementary material

Kiely et al. supplementary material 5 Kiely et al. supplementary material

Kiely et al. supplementary material 6 Kiely et al. supplementary material

## Data Availability

The data-sets generated and analysed for this study can be obtained from the authors on request.

## Footnotes

a. The term ‘longstanding’ has been put forth as an alternative to SEED, affirmed by voices of lived experience. Further studies could confirm the ‘epistemic justice’ of this term as well as the diagnostic construct. Thus, the term longstanding anorexia nervosa (L-AN) is respectfully adopted for this paper, until such time of consensus on the most acceptable terminology.

b. Note: information about comorbidity and other psychiatric history was collected only in the second iteration of the EDTES distributed in 2022.

## Supplementary material

The supplementary material is available online at https://doi.org/10.1192/bjo.2025.10923
