# Supplementary material for: Key features of illness and treatment experiences in longstanding anorexia nervosa: qualitative descriptive study
Source: BJPsych Open. 2025 Dec 22;12(1):e22. doi: 10.1192/bjo.2025.10923 (PMC12724101; doi:10.1192/bjo.2025.10923)
Supplement: Kiely et al. supplementary material 1 — Kiely et al. supplementary material [file S205647242510923Xsup001.pdf]

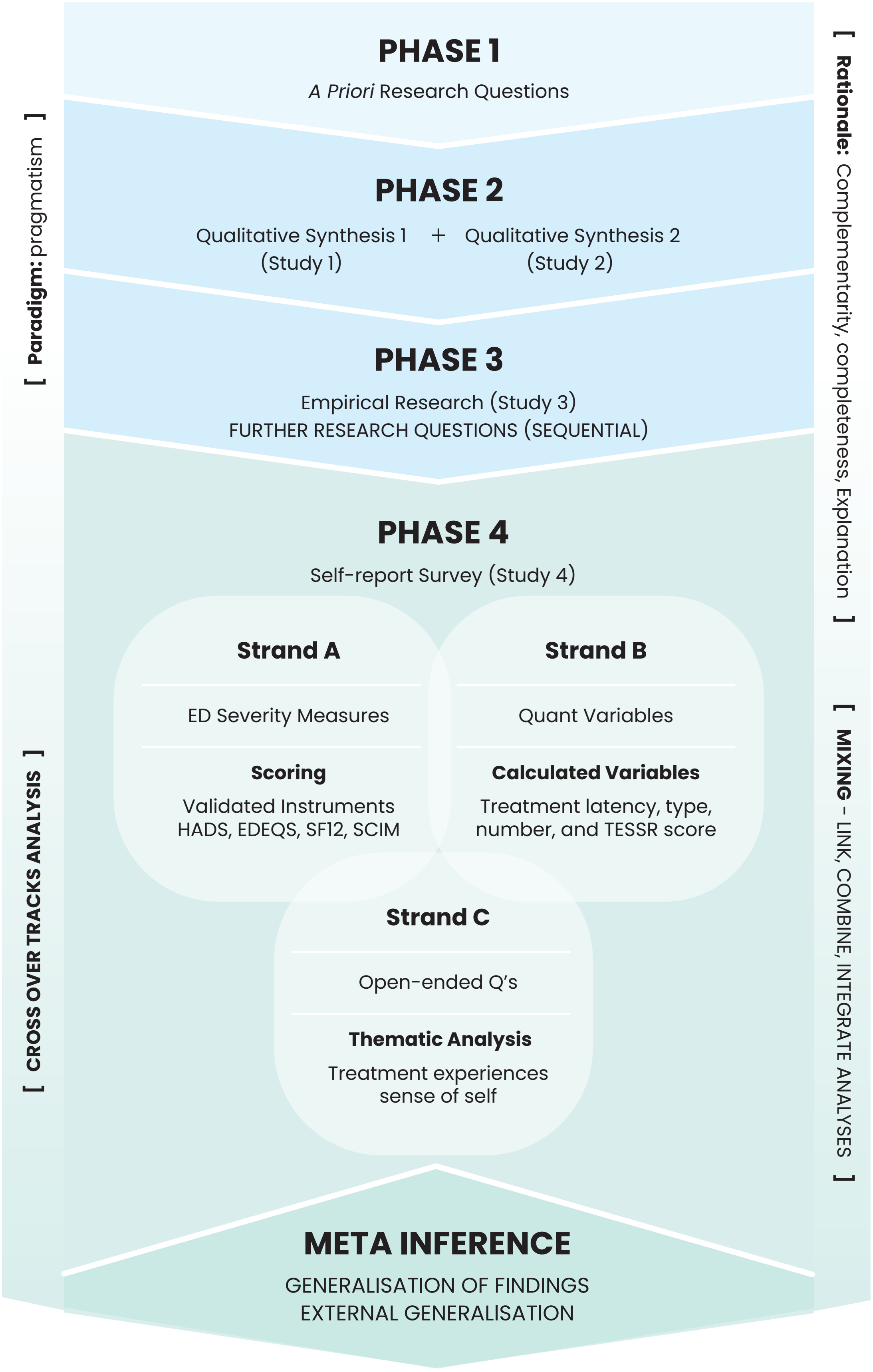


*HADS=hospital anxiety and depression score; EDEQS=eating disorder examination questionnaire (short); SF12= health related quality of life short form 12 version 2; SCIM= Self-concept and identity measure; TESSR=treatment experiences sliding scale rater*
