# Supplementary material for: Key features of illness and treatment experiences in longstanding anorexia nervosa: qualitative descriptive study
Source: BJPsych Open. 2025 Dec 22;12(1):e22. doi: 10.1192/bjo.2025.10923 (PMC12724101; doi:10.1192/bjo.2025.10923)
Supplement: Kiely et al. supplementary material 2 — Kiely et al. supplementary material [file S205647242510923Xsup002.pdf]

Supplementary File 2: STROBE Statement and COREQ_Checklist

STROBE Statement—Checklist of items that should be included in reports of ***cross-sectional studies***

	Item No	Recommendation	Tick /pg. #
**Title and abstract**	1	(*a*) Indicate the study's design with a commonly used term in the title or the abstract	✓
		(*b*) Provide in the abstract an informative and balanced summary of what was done and what was found	✓
**Introduction**			
Background/rationale	2	Explain the scientific background and rationale for the investigation being reported	3
Objectives	3	State specific objectives, including any prespecified hypotheses	7
**Methods**			
Study design	4	Present key elements of study design early in the paper	7
Setting	5	Describe the setting locations, and relevant dates, including periods of recruitment , exposure, follow-up, and data collection YES	8
Participants	6	(*a*) Give the eligibility criteria, and the sources and methods of selection of participants	8
Variables	7	Clearly define all outcomes, exposures, predictors, potential confounders, and effect modifiers. Give diagnostic criteria, if applicable	8
Data sources/ measurement	8*	For each variable of interest, give sources of data and details of methods of assessment (measurement). Describe comparability of assessment methods if there is more than one group	9-11
Bias	9	Describe any efforts to address potential sources of bias	11
Study size	10	Explain how the study size was arrived at	
Quantitative variables	11	Explain how quantitative variables were handled in the analyses. If applicable, describe which groupings were chosen and why	12
Statistical methods	12	(*a*) Describe all statistical methods, including those used to control for confounding	12
		(*b*) Describe any methods used to examine subgroups and interactions	12
		(*c*) Explain how missing data were addressed	12
		(*d*) If applicable, describe analytical methods taking account of sampling strategy	n/a
		(*e*) Describe any sensitivity analyses	n/a
**Results**			
Participants	13*	(a) Report numbers of individuals at each stage of study—eg numbers potentially eligible, examined for eligibility, confirmed eligible, included in the study, completing follow-up, and analysed	13 (and tables)

		(b) Give reasons for non-participation at each stage	Tick /pg. #
		(c) Consider use of a flow diagram	
Descriptive data	14*	(a) Give characteristics of study participants (eg demographic, clinical, social) and information on exposures and potential confounders	✓
		(b) Indicate number of participants with missing data for each variable of interest	✓
Outcome data	15*	Report numbers of outcome events or summary measures	
Main results	16	(*a*) Give unadjusted estimates and, if applicable, confounder-adjusted estimates and their precision (eg, 95% confidence interval). Make clear which confounders were adjusted for and why they were included	n/a
		(*b*) Report category boundaries when continuous variables were categorized	
		(*c*) If relevant, consider translating estimates of relative risk into absolute risk for a meaningful time period	n/a
Other analyses	17	Report other analyses done—eg analyses of subgroups and interactions, and sensitivity analyses	12
**Discussion**			
Key results	18	Summarise key results with reference to study objectives	24
Limitations	19	Discuss limitations of the study, taking into account sources of potential bias or imprecision. Discuss both direction and magnitude of any potential bias	29
Interpretation	20	Give a cautious overall interpretation of results considering objectives, limitations, multiplicity of analyses, results from similar studies, and other relevant evidence	29
Generalisability	21	Discuss the generalisability (external validity) of the study results	29
**Other information**			
Funding	22	Give the source of funding and the role of the funders for the present study and, if applicable, for the original study on which the present article is based	n/a

*Give information separately for exposed and unexposed groups.

Note: An Explanation and Elaboration article discusses each checklist item and gives methodological background and published examples of transparent reporting. The STROBE checklist is best used in conjunction with this article (freely available on the Web sites of PLoS Medicine at http://www.plosmedicine.org/, Annals of Internal Medicine at http://www.annals.org/, and Epidemiology at http://www.epidem.com/). Information on the STROBE Initiative is available at www.strobe-statement.org.

COREQ (COnsolidated criteria for REporting Qualitative research) Checklist

A checklist of items that should be included in reports of qualitative research. You must report the page number in your manuscript where you consider each of the items listed in this checklist. If you have not included this information, either revise your manuscript accordingly before submitting or note N/A.

Topic	Item No.	Guide Questions/Description	Reported on Page No.
**Domain 1: Research team and reflexivity**		*PLEASE NOTE: THIS RESEARCH INCLUDED QUALITATIVE ANALYSIS OF TEXT RESPONSES NOT AN INTERVIEW*	
Personal characteristics		please refer to additional online file 3 for information re: personal characteristics	
Interviewer/facilitator	1	Which author/s conducted the interview? ***pertains to text thematic analysis***	ALL see pg. 11
Credentials	2	What were the researcher's credentials? E.g. PhD, MD	PHD/ PROFESS
Occupation	3	What was their occupation at the time of the study?	PSYCHOLOGY/
Gender	4	Was the researcher male or female?	FEMALE
Experience and training	5	What experience or training did the researcher have?	PHD CANDIDA
Relationship with participants			
Relationship established	6	Was a relationship established prior to study commencement?	NO
Participant knowledge of the interviewer	7	What did the participants know about the researcher? e.g. personal goals, reasons for doing the research	NONE
Interviewer characteristics	8	What characteristics were reported about the inter viewer/facilitator? e.g. Bias, assumptions, reasons and interests in the research topic	REFLECTIVE ST
**Domain 2: Study design**			
Theoretical framework			
Methodological orientation and Theory	9	What methodological orientation was stated to underpin the study? e.g. grounded theory, discourse analysis, ethnography, phenomenology, content analysis	GT/PHENOMEN see pages 7&8
Participant selection			
Sampling	10	How were participants selected? e.g. purposive, convenience, consecutive, snowball	SNOWBALL pg
Method of approach	11	How were participants approached? e.g. face-to-face, telephone, mail, email	INTERNET pg.1
Sample size	12	How many participants were in the study?	208 pg 13
Non-participation	13	How many people refused to participate or dropped out? Reasons?	some did not r
Setting			
Setting of data collection	14	Where was the data collected? e.g. home, clinic, workplace	online pg 12
Presence of non-participants	15	Was anyone else present besides the participants and researchers?	n/a
Description of sample	16	What are the important characteristics of the sample? e.g. demographic data, date	worldwide pg
Data collection			
Interview guide	17	Were questions, prompts, guides provided by the authors? Was it pilot tested?	n/a
Repeat interviews	18	Were repeat inter views carried out? If yes, how many?	n/a
Audio/visual recording	19	Did the research use audio or visual recording to collect the data?	n/a
Field notes	20	Were field notes made during and/or after the inter view or focus group?	n/a
Duration	21	What was the duration of the inter views or focus group?	n/a
Data saturation	22	Was data saturation discussed?	yes
Transcripts returned	23	Were transcripts returned to participants for comment and/or	n/a

Topic	Item No.	Guide Questions/Description	Reported on Page No.
		correction?	
**Domain 3: analysis and findings**			
Data analysis			
Number of data coders	24	How many data coders coded the data?	1 pg. 11
Description of the coding tree	25	Did authors provide a description of the coding tree?	table S1 & Fig [illegible]
Derivation of themes	26	Were themes identified in advance or derived from the data?	derived eg pg[illegible]
Software	27	What software, if applicable, was used to manage the data?	excel
Participant checking	28	Did participants provide feedback on the findings?	no
Reporting			
Quotations presented	29	Were participant quotations presented to illustrate the themes/findings? Was each quotation identified? e.g. participant number	yes tables and[illegible]
Data and findings consistent	30	Was there consistency between the data presented and the findings?	yes
Clarity of major themes	31	Were major themes clearly presented in the findings?	yes
Clarity of minor themes	32	Is there a description of diverse cases or discussion of minor themes?	no

Developed from: Tong A, Sainsbury P, Craig J. Consolidated criteria for reporting qualitative research (COREQ): a 32-item checklist for interviews and focus groups. *International Journal for Quality in Health Care*. 2007. Volume 19, Number 6: pp. 349 – 357

Once you have completed this checklist, please save a copy and upload it as part of your submission. DO NOT include this checklist as part of the main manuscript document. It must be uploaded as a separate file.
