# Supplementary material for: Key features of illness and treatment experiences in longstanding anorexia nervosa: qualitative descriptive study
Source: BJPsych Open. 2025 Dec 22;12(1):e22. doi: 10.1192/bjo.2025.10923 (PMC12724101; doi:10.1192/bjo.2025.10923)
Supplement: Kiely et al. supplementary material 3 — Kiely et al. supplementary material [file S205647242510923Xsup003.docx]

**Eating Disorder Examination Questionnaire (Short) – EDE-QS**

For the EDE-QS, we chose to include the qualifier ‘a lot’ for the term ‘markedly’. Questions relate to eating (restriction and loss of control), compensatory behaviors, cognitive preoccupations, as well as drive for thinness, fear, and body dissatisfaction. While a score of 15 is considered symptomatic of an ED (1) for screening purposes, a lower score, in the presence of eating disorder behaviors (restriction, binge eating, and compensation), represents clinically impactful AN symptomatology (2). In the present study, the internal consistency was 0.868 (Cronbach’s α) for the 12 items.

**Self-Concept and Identity Measure (SCIM)**

The measure is empirically validated in clinical samples (3) and has good internal reliability for both composite and subscores (α = 0.86; α = 0.73 to 0.86), with good retest reliability (3). From a seven-point agreement scale, a higher total score (out of a possible 189) correlates with greater identity disturbance and psychopathology (3, 4). For this research, we chose to study composite scores of identity disturbance only. A total SCIM score of 91.49 (26.58) has been reported in a sample of 74 women (mean age 28.26 years) with a current AN diagnosis of unspecified duration(5). A clinical sample of 216 female (median age 35) treatment-seekers for substance misuse with comorbid depression, PTSD, and eating disorders (3) had comparatively lower total average scores of 74.80 (19.85). The internal consistency of the measure for the present sample was Cronbach’s α of 0.904.

**Health-Related Quality of Life – Short Form 12 version (SF12v2)**

SF12-v2 (6) categorizes a person’s physical component score (PCS) and mental component score (MCS) as “Same or Better,” “Below,” or “Well Below” benchmarked scores, compared to the general population (aged 18-76 years). Numerically, a lower score further from a mean of 50 indicates poorer HR-QoL. The score has demonstrated good internal consistency (Cronbach’s α PCS = 0.87 and MCS = 0.82) (7) and construct validity, as well as cross-cultural sensitivity (8). In the present study, Cronbach’s α was 0.909 for the 12 items.

**Hospital Anxiety and Depression Score (HADS)**

It is widely validated in healthy and clinical adult populations internationally, across the lifespan. A score of >10 is pathological for either depression or anxiety (9). Given its relative age (i.e., 1983), some nouns were replaced to be more contemporary (e.g., “listening to a Podcast or audiobook” instead of “radio”). In the present study, the internal consistency, as measured by Cronbach’s α, was 0.671 for the 14 items.

**Treatment Experiences Sliding Scale Rater (TESSR)**

The TESSR (10) explores several domains of the treatment experience related to perceived freedom of choice, preferences, therapeutic relationship, readiness to change, hope, and perceived overall helpfulness. A copy of the instrument is included below. For example, participants were asked “*Overall, how helpful do you think this eating disorder treatment was?*” and responded via a sliding scale: “*not helpful at all*” (-50) to “*extremely helpful*” (+50). To further contextualize the findings, the written descriptions of L-AN participants’ treatments were categorized by treatment type (e.g., outpatient CBT or residential ED program). The internal consistency for this measure (Cronbach’s α) in the present sample for the 11 most and least helpful items was 0.908 and 0.902, respectively. TESSR has been found to have high internal consistency (for most helpful items α = 0.926 and for least helpful items α = 0.868) (10).

**The Treatment Experiences Sliding Scale Rater (TESSR) © instrument**

**Ten questions for perceived least and most helpful treatment experiences**

**Treatment Experiences Sliding Scale Rater (TESSR) ©**

1. When I sought this treatment

|  | I did NOT think change was important or possible | I thought change was important or possible |
| --- | --- | --- |

|  | 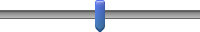 |
| --- | --- |

1. The treatment approach

|  | Did NOT work for me | Worked well for me |
| --- | --- | --- |

|  | 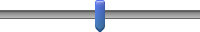 |
| --- | --- |

1. The treatment approach

|  | Did NOT meet my hopes and expectations | Met my hopes and expectations |
| --- | --- | --- |

|  | 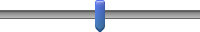 |
| --- | --- |

1. The treatment approach

|  | Did NOT assist me in shifting my relationship with difficult emotions | Assisted me in shifting my relationship with difficult emotions |
| --- | --- | --- |

|  | 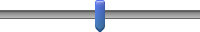 |
| --- | --- |

1. The treatment approach

|  | Did NOT assist me in recovering from the eating disorder | Assisted me in recovering from the eating disorder |
| --- | --- | --- |

|  | 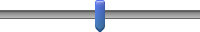 |
| --- | --- |

1. The treatment approach

|  | Did NOT take into consideration my treatment preferences | Took into consideration my treatment preferences |
| --- | --- | --- |

|  | 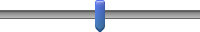 |
| --- | --- |

1. The treatment approach

|  | Did NOT give me freedom to make my own choices around change | Gave me freedom to make my own choices around change |
| --- | --- | --- |

|  | 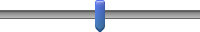 |
| --- | --- |

1. The treatment approach

|  | I did NOT feel understood by the therapist | I felt understood by the therapist |
| --- | --- | --- |

|  | 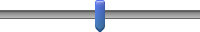 |
| --- | --- |

1. The treatment approach

|  | The therapist did NOT address my concerns | The therapist addressed my concerns |
| --- | --- | --- |

|  | 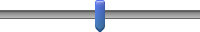 |
| --- | --- |

1. The treatment approach

|  | The therapist DID NOT instil hope for recovery | The therapist DID instil hope for recovery |
| --- | --- | --- |

|  | 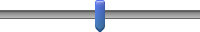 |
| --- | --- |

1. Overall, how helpful do you think your eating disorder treatment was?

|  | Not helpful at all | Extremely helpful |
| --- | --- | --- |

|  | 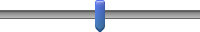 |
| --- | --- |

Questions 1-10 utilise a Likert sliding scale response ( -50 to 50, range= 100)

© The Treatment Experiences Sliding Scale Rater (TESSR) can be used and reproduced with permission from the author: J.Conti@westernsydney.edu.au

**Data Analysis – Statistical Analyses**

Quantitative statistical methods (correlations and between-group comparisons) were employed to test the presence of putative defining features related to the currently proposed definition of L-AN. IBM® SPSS® Statistics 26 was used for all statistical analyses. Data were exported from Qualtrics, inspected, cleaned, and tested for normality. Nonparametric descriptives and continuous data utilized the median and interquartile range (IQR), while for categorical data, the mean and standard deviation, or N%, were used. Inferential statistics, e.g., correlation coefficient (Spearman r_s_) were used to explore associations between the putative features of L-AN and DoI. Between-group comparisons employed univariate statistical tests as appropriate, such as the t-test or Mann-Whitney U test^[[1]](#footnote-1)^ for continuous data, or the Chi-square (χ^2^) test for categorical data. The Mann-Whitney U test was also used when sample sizes were less than 10 as a more conservative approach. In the case of underpowered samples, correlations were not reported. No imputations were made for responses that were missing. Statistical significance was set at p<0.05, and Cronbach’s α was used to assess the reliability of quantitative measures.

We did not have pilot data on which to base an *a priori* power estimate. However, regarding the statistics in Table 3, for the parametric t-test, according to Cohen’s estimate, for a power of 0.2 and a one-sided alpha of 0.05, the minimum sample size per group is 21 for a standardized effect size (ES) of 0.8 (a large effect size) and 51 for a medium ES. We have added Cohen’s *d* to Table 3. We have conducted a retrospective power analysis for the Mann-Whitney U tests, with a 95% probability of preventing Type 1 error and 80% probability of preventing Type 2 error. For MHRQoL, the minimum sample size should be 58 per group; thus, we acknowledge that we were underpowered.^[[2]](#footnote-2)^

Regarding the statistics in Table 4, for Spearman rank (rs) correlational statistics (11) with 95% confidence interval (CI) and medium precision (CI width of 0.3), the recommended sample size is between 35 (for rs = 0.8) and 111 (for rs = 0.3). Thus, power was adequate for the Spearman rank correlations (sample n=199). Regarding Table 5, we only presented statistics for groups with a sample size greater than 30. The post-hoc power calculation for an alpha of 0.05 was 5.6%.

1. Prnjak K, Mitchison D, Griffiths S, Mond J, Gideon N, Serpell L, et al. Further development of the 12-item EDE-QS: identifying a cut-off for screening purposes. BMC psychiatry. 2020;20(1):146-.DOI: 10.1186/s12888-020-02565-5.

2. Christian C, Williams BM, Hunt RA, Wong VZ, Ernst SE, Spoor SP, et al. A network investigation of core symptoms and pathways across duration of illness using a comprehensive cognitive–behavioral model of eating-disorder symptoms. Psychological medicine. 2021;51(5):815-24.DOI: 10.1017/S0033291719003817.

3. Kaufman EA, Puzia ME, Crowell SE, Price CJ. Replication of the Self-Concept and Identity Measure (SCIM) Among a Treatment-Seeking Sample. Identity (Mahwah, NJ). 2019;19(1):18-28.DOI: 10.1080/15283488.2019.1566068.

4. Bogaerts A, Claes L, Verschueren M, Bastiaens T, Kaufman EA, Smits D, et al. The Dutch Self-Concept and Identity Measure (SCIM): Factor structure and associations with identity dimensions and psychopathology. Personality and Individual Differences. 2018;123:56-64.DOI: <https://doi.org/10.1016/j.paid.2017.11.007>.

5. Croce SR, Malcolm AC, Ralph‐Nearman C, Phillipou A. Examining Identity Functioning in Anorexia Nervosa Across Illness and Recovery Stages. The International journal of eating disorders. 2024;57(9):1959-68.DOI: 10.1002/eat.24247.

6. Ware JE, Kosinski M, Keller SD. A 12-Item Short-Form Health Survey: Construction of Scales and Preliminary Tests of Reliability and Validity. Medical care. 1996;34(3):220-33.DOI: 10.1097/00005650-199603000-00003.

7. Montazeri A, Vahdaninia M, Mousavi SJ, Asadi-Lari M, Omidvari S, Tavousi M. The 12-item medical outcomes study short form health survey version 2.0 (SF-12v2): a population-based validation study from Tehran, Iran. Health and quality of life outcomes. 2011;9(1):12-.DOI: 10.1186/1477-7525-9-12.

8. Lam ETP, Lam CLK, Fong DYT, Huang WW. Is the SF-12 version 2 Health Survey a valid and equivalent substitute for the SF-36 version 2 Health Survey for the Chinese? Journal of evaluation in clinical practice. 2013;19(1):200-8.DOI: 10.1111/j.1365-2753.2011.01800.x.

9. Vodermaier A, Millman RD. Accuracy of the Hospital Anxiety and Depression Scale as a screening tool in cancer patients: a systematic review and meta-analysis. Supportive care in cancer. 2011;19(12):1899-908.DOI: 10.1007/s00520-011-1251-4.

10. Mital R, Hay P, Conti JE, Mannan H. Associations between therapy experiences and perceived helpfulness of treatment for people with eating disorders. Journal of eating disorders. 2022;10(1):80-.DOI: 10.1186/s40337-022-00601-1.

11. Mohamad Adam B. An elaboration on sample size determination for correlations based on effect sizes and confidence interval width: a guide for researchers. Restorative dentistry & endodontics. 2024;49(2):21.1-.8.

1. See Benchmark Six Sigma. Sample Size Calculator for Mann Whitney Test. 2025 https://www.benchmarksixsigma.com/calculators/sample-size-calculator-for-mann-whitney-test/ [↑](#footnote-ref-1)
2. See ClinCalc. Post-hoc Power Calculator. 2025. https://clincalc.com/stats/power.aspx [↑](#footnote-ref-2)
