# Supplementary material for: Key features of illness and treatment experiences in longstanding anorexia nervosa: qualitative descriptive study
Source: BJPsych Open. 2025 Dec 22;12(1):e22. doi: 10.1192/bjo.2025.10923 (PMC12724101; doi:10.1192/bjo.2025.10923)
Supplement: Kiely et al. supplementary material 4 — Kiely et al. supplementary material [file S205647242510923Xsup004.doc]

**Supplementary 4: Process of thematic analysis**

Strand C – Qualitative: Thematic Analyses

One author (LK) extracted the data, immersing themselves in the content to develop a big picture understanding of the collective responses (Phase 1). Individual responses were then interpreted and paraphrased line by line (Phase 2) to distinguish the salient features. Descriptive codes (Phase 3) were allocated, and conferring on interpretations and preliminary codes occurred in Phase 4 with a second author (JC). The codes were then grouped and consolidated via an iterative process (Phase 5), and overarching “meta” and “sub” themes were generated (all authors). In Phase 6, the authors returned to the raw extractions and color-coded the participant responses according to the themes to cross-check and create a directory of participant quotes.

**Supplementary 4: Thematic analysis of open-ended responses: self-concept and identity**

| **THEME 1**  **Disconnected self**  *‘I wasn’t a real person’* | **DESCRIPTION** | **EXEMPLARS** |
| --- | --- | --- |
|  | Participant extracts illustrated a loss of connection to self through experiences such as dissociation, shame, and a fractured sense of self that inter-related with the ED experience | **Participant 40:** *While I was quite unwell with my ED I had a strong sense of feeling like* ***I wasn’t a “real person****”, wasn’t normal and was* ***somehow broken*** *and* ***unable to be fixed***  **Participant 13:** *I am not the same person. The* ***persona*** *I experienced* ***in the height of the disorder*** *is completely different 5 years into recovery*  **Participant 7:** *I* ***often feel numb*** *&* ***like I’m not real****. I know who I used to be and I know I still value the same things but I feel as though it’s* ***cloudy*** *when* ***trying to remember & become that person***  **Participant 38: *I feel like an empty person***  **Participant 10:** *I used to feel so* ***vile*** *and abhorrent as a person that* ***at my lowest points****, I was actually startled momentarily when bright red blood appeared in a blood test or similar. The* ***disgust and*** ***shame*** *I* ***felt to my core*** ***causing me to starve to death*** *meant I surely had a* ***putrid black sludge infiltrating my body*** *in place of blood****; that I must be as disgusting inside*** *as I felt on the outside.* |
| **Theme 2a**.  **Self and recovery processes:** Making Sense of Self and AN | Central to recovery accounts was understanding the purpose of the AN in their life and in increasing self-understanding. This assisted in an iterative recovery which included coherent sense of self that encapsulated what they learnt about themselves through the AN experience | **Participant 41:** *My ED has always been* ***interconnected with my autism****….The diagnosis changed so much about* ***how I see myself as a person*** *- it made sense of why I've* ***always felt different*** *or* ***a bit wrong****, why and how I've learnt to camouflage and adapt to the people around me, and how* ***this eroded a solid sense of self***  **Participant 26:** *My eating disorder* ***shone a light of family dysfunction*** *.. enabled me to work towards the root cause… I only wish that as an adult that my ED had been seen as the* ***symptom of family dysfunction and ASD***  **Participant 23:** *I experienced* ***childhood trauma*** *and have now been in* ***therapy for 7 years*** *(at the time I was heavily in my active eating disorder). My responses would have been different pre therapy.*  **Participant 43:**  *The* ***ED has taught me*** *there are so many* ***layers*** *to me, and I need to* ***understand what lies beneath*** *before I can hope to catch many of the behaviours that plague me despite treatment.... until I* ***address the noise that is ME****, nothing [in treatment] is ever going to make a difference*  **Participant 47***: I’ve learned that* ***hiding from myself*** *and* ***from the real world*** *in the* ***AN bubble*** *for 50+ years makes it very difficult to navigate life in the real world* |
| **Theme 2b.**  **Self and recovery processes:** Disentangling self and healing shame | Recovering a sense of self meant integrating the ED experience into a coherent sense of self and making meaning of those experiences rather than rejecting the ED dichotomously | **Participant 36:** *The eating disorder has ended up becoming a very prominent part of my life, even now from a recovery-oriented perspective and helping others who are struggling.... both are equally me, and for the majority of my life it's felt that they were opposites. I am now trying to bridge those gaps which is why I feel less "broken."*  **Participant 19:** *A lot of my “weaknesses” and vulnerabilities are also my greatest strengths and most defining parts of who I am. The world, however, is quick to judge and point blame at certain characteristics simply because they are assumed to have led to an ED. But had they been channelled proactively, maybe that wouldn’t have been the case. If someone had tried to really understand my grievances, perhaps I would have felt “human” instead of “broken”.*  **Participant 10***: Recovery itself is an incredible way of being forced to value yourself. I don't know that I would've faced myself otherwise. It's a gift in a way*  **Participant 39:** *the healthy part of me knows strongly who I am and doesn’t think I’m a horrible person. However, my eating disorder and inner critic are very loud and have a different believe about me*  **Participant 1:** *I don’t know if my beliefs and thoughts are my own or the disorders*  **Participant 10:** *And yet after decades of anorexia, I emerged on the other side. knowing with complete certainty that I am* ***a good person,*** ***a person who deserves wonderful things*** *as much as anyone else ♡xx* |
| **Theme 3a.**  **Cross cutting theme:**  *It took time* | AN was described as complex and therefore it took time for treatment and to find the right treatment that could assist a person to understand and make meaning of the AN | **Participant 3:** *I am* ***several years*** *into a* ***recovery journey****. My eating disorder began over 20 years ago and* ***only in the past 3 years*** *have I been able to significantly reduce behaviors. It has been a* ***long,*** *arduous journey, but the work is paying off*  **Participant 23***: I experienced childhood trauma and have now been in therapy for 7 years*  **Participant 5***: for a while I had to become a carer* ***for the person I was,*** *to be able to become* ***the person I wanted to be, it took time*** *but I am at a healthy weight.*  **Participant 29*:*** *This psychologist helped me address multiple underlying issues behind the eating disorder and we* ***worked on each issue in stages*** *over the past 5+ years*  **Participant 70:** *It was helpful because* ***I was there long term*** *and got to* ***build trusting*** *relationships with my whole treatment team*  **Participant 166**: ..*were compassionate and* *built a stable foundation by working through the trauma that made me this way and built up on that and* ***challenged me in my own pace,*** *while* ***slowly*** *getting me out of my comfort zone.*  **Participant 122:** *one in one therapy many years later… Worked through the reasons I developed the disorder, worked on self esteem and empowerment*  **Participant 96:** *It was* ***very short****. The mental health team I was under believed I did not need long term 1 -to-1 treatment however, they* ***did not ask my views on this***  **Participant 90: *Long term help****…The* ***constant support*** *and help* |
| **Theme 3b***.*  **Cross cutting theme:**  *Treatment harm and limitations* | Participants reflected on some of the challenges they experienced with treatments that inadvertently further contributed to a vulnerable sense of self, including an internalisation of the problem within them – where they were positioned as the problem. Treatment limitations protracted the recovery process and at times treatment was experienced as traumatic. | **Participant 23***: I don’t really feel like a person at all.* ***ED treatment never helped*** *because* ***when something didn't work/resonate it felt like there was something wrong with me****, and* ***if I were a proper/complete person it would have been a better fit***  **Participant 26:**  *I only wish that as an adult that my ED had been seen as the symptom of these things, family issues and neurodiversity. But now I know and I can do better, even though many ED treatments do not, ie CBT, etc.*  **Participant 8***: It's tough to put into words. Sometimes I feel like the* ***treatment trauma*** *I went through made me a stronger person more than the treatment itself*  **Participant 11***: I think fighting for my sense of self got caught up with fighting to maintain the eating disorder and this led to treatment being ineffective*  **Participant 4 1** *my* ***autism****… was* ***undiagnosed*** *until I was 36 years old, 18 years after my disordered eating began, and* ***9 or so years after I started treatment****.*  **Participant 38:…***but I think I'm more sure of who I am after doing DBT* |
