# Supplementary material for: Key features of illness and treatment experiences in longstanding anorexia nervosa: qualitative descriptive study
Source: BJPsych Open. 2025 Dec 22;12(1):e22. doi: 10.1192/bjo.2025.10923 (PMC12724101; doi:10.1192/bjo.2025.10923)
Supplement: Kiely et al. supplementary material 5 — Kiely et al. supplementary material [file S205647242510923Xsup005.docx]

**Supplementary 5: Authors’ reflective statements**

Of relevance for this review, Laura Kiely (LK) has worked clinically with people experiencing eating disorders (ED), encompassing end-stage care, as an accredited practicing dietitian and professionally registered psychotherapist/counsellor, specializing in Gestalt therapy (GT) interventions. GT fits within an existential, relational paradigm, which informed the analysis. LK has witnessed and personally experienced the limitations of current treatment paradigms and the systemic inadequacies for those with EDs, including SE-AN. This was the impetus for the doctoral research, of which the present paper forms one chapter. Many layers of supervision – research supervision, clinical, peer, and group supervision – supported the first author to at times put aside and at other times expand assumptions to remain true to the phenomenological method and ensure gaps were addressed in the hermeneutic process with additional triangulation between researchers. LK has their own recovery experience.

Janet Conti (JC) is a Clinical Psychologist and academic in Clinical Psychology who started her work with people who experience EDs as a dietitian. Her research and clinical work are informed by the paradigm of narrative therapy (92) and seek to prioritize the voice of the experiencing person to inform the development of a broader range of ED treatment interventions that have scope to be flexibly tailored to the needs and preferences of the experiencing person and their family.

Phillipa Hay (PH) is a Clinical Academic Psychiatrist, formally trained first in psychodynamic psychotherapy and then in cognitive behavior therapy. She has experience in caring for many people with L-AN in general hospitals and outpatient private practice settings, was a lead investigator on a L-AN clinical trial (19), and lead author on Australian guidelines (93) endorsing the need for new person-centered and flexible approaches in care.
