# Supplementary material for: Key features of illness and treatment experiences in longstanding anorexia nervosa: qualitative descriptive study
Source: BJPsych Open. 2025 Dec 22;12(1):e22. doi: 10.1192/bjo.2025.10923 (PMC12724101; doi:10.1192/bjo.2025.10923)
Supplement: Kiely et al. supplementary material 6 — Kiely et al. supplementary material [file S205647242510923Xsup006.docx]

**Supplementary 6: List of open-ended response survey questions analyzed as part of the qualitative analysis**

The Eating Disorder and Treatment Experiences Survey (EDTES) asked participants the following questions:

*Are there any other comments you would like to share with us about any aspect of this survey (e.g., your eating disorder and/or treatment experiences, feedback)?*

We will now ask you some questions about the eating disorder treatment you feel was least/ most helpful to you. Please try to answer all questions in a way that best reflects your memory of this treatment experience.

*The eating disorder treatment that I experienced that was LEAST/ MOST helpful is/was: (open-ended description)*

*Please tell us more about the eating disorder treatment that was most/least helpful for you. (For example, what was it, why do you feel this was the most helpful? What made this different from your other eating disorder treatment experiences?)*

*What was the most/least helpful aspect about this treatment, and why?*

*What was least helpful aspect about this treatment, and why?*

*Based on your experience what would you have liked more of in this least/ most helpful treatment?*

*Was there anything that was helpful about this least helpful treatment? If so, please explain what was helpful?*

The SCIM supplementary questions were designed to distill information about self-perceptions and the perceived impact of the eating disorder experience on the sense of self. The questions were:

*Do you have any comments on what was it like for you to complete this survey? Please share*

*Is there anything else you would like us to know about how you see yourself as a person, what you have learnt about yourself as a person through your experience of an eating disorder.*
